# Long trimer-immunization interval and appropriate adjuvant reduce immune responses to the soluble HIV-1-envelope trimer base

**DOI:** 10.1016/j.isci.2024.108877

**Published:** 2024-01-11

**Authors:** Hongying Duan, Angela R. Corrigan, Cheng Cheng, Andrea Biju, Christopher A. Gonelli, Adam S. Olia, I-Ting Teng, Kai Xu, Sijy O’Dell, Sandeep Narpala, Mike Castro, Leonid Serebryannyy, Jennifer Wang, Danealle K. Parchment, Edward K. Sarfo, Jelle van Schooten, John-Paul Todd, Shuishu Wang, Darcy R. Harris, Hui Geng, Alexander J. Jafari, VRC Production Program, Ruth A. Woodward, Nicole A. Doria-Rose, Kathryn E. Foulds, Adrian B. McDermott, Marit J. van Gils, Richard A. Koup, Theodore C. Pierson, Peter D. Kwong, John R. Mascola

**Affiliations:** 1 Vaccine Research Center, National Institutes of Allergy and Infectious Diseases, National Institutes of Health, Bethesda, MD 20892, USA; 2 Department of Medical Microbiology, Amsterdam UMC, University of Amsterdam, Amsterdam Institute for Infection and Immunity, Amsterdam 1105AZ, the Netherlands

**Keywords:** Immune response, Virology, Immunology

## Abstract

Soluble ‘SOSIP’-stabilized HIV-1 envelope glycoprotein (Env) trimers elicit dominant antibody responses targeting their glycan-free base regions, potentially diminishing neutralizing responses. Previously, using a nonhuman primate model, we demonstrated that priming with fusion peptide (FP)-carrier conjugate immunogens followed by boosting with Env trimers reduced the anti-base response. Further, we demonstrated that longer immunization intervals further reduced anti-base responses and increased neutralization breadth. Here, we demonstrate that long trimer-boosting intervals, but not long FP immunization intervals, reduce the anti-base response. Additionally, we identify that FP priming before trimer immunization enhances antibody avidity to the Env trimer. We also establish that adjuvants Matrix M and Adjuplex further reduce anti-base responses and increase neutralizing titers. FP priming, long trimer-immunization interval, and an appropriate adjuvant can thus reduce anti-base antibody responses and improve Env-directed vaccine outcomes.

## Highlights

• We identify factors that improve or impact Env-directed HIV-1-vaccine outcomes
• Long Env trimer interval, but not long FP interval, reduces anti-base responses
• FP priming prior to trimer immunization increases antibody avidity to trimer
• Adjuvants impact anti-base and epitope-specific immune responses

## Graphical abstract

**Figure:**
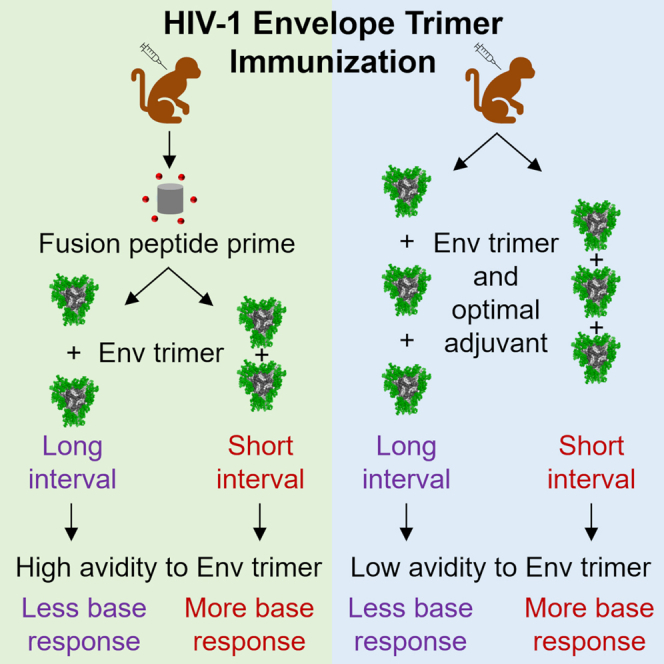

## Introduction

The HIV-1 envelope glycoprotein (Env) trimer, comprising gp120 and gp41 subunits, is the sole virally encoded surface protein on the HIV-1 virion, making it a target for vaccine design. Soluble Env trimers from multiple HIV-1 strains have been stabilized by the introduction of an inter-subunit disulfide bond A501C/T605C (SOS) to covalently link gp120 to gp41 and an isoleucine to proline mutation (IP) to create vaccine candidates that maintain the presentation of critical epitopes with native-like glycosylation, in a prefusion-closed conformation.^1^^,^^2^ Immunization with stabilized soluble trimers elicits autologous neutralization activity in mice, guinea pigs, rabbits, and nonhuman primates (NHPs); such immunizations, however, generally fail to elicit robust heterologous titers.^3^^,^^4^^,^^5^^,^^6^^,^^7^ Strategies to improve neutralization activity from trimer immunogens have been explored, such as removing or adding glycans to the soluble trimers to drive responses toward key epitopes such as the CD4-binding or fusion peptide (FP) sites of vulnerability,^8^^,^^9^^,^^10^^,^^11^^,^^12^ performing sequential immunization with several SOSIP-trimers of different clades,^9^^,^^13^ or incorporating additional trimer-stabilizing mutations.^1^^,^^14^^,^^15^^,^^16^^,^^17^ However, these strategies have been suboptimal, in part, due to the exposure of a highly accessible and immunogenic glycan-free base present on these trimers. Dominant trimer-elicited non-neutralizing anti-base antibody responses have been observed in preclinical animal models^3^^,^^4^^,^^5^^,^^6^^,^^7^ and human clinical trials.^18^ Additionally, an overwhelming anti-base antibody response can render trimers susceptible to degradation, with the resulting protomers inducing additional off-target antibody responses.^19^ Novel trimer immunogens and immunization regimens that limit the elicitation of anti-base antibody responses may be essential to improve vaccine-induced neutralization activity and breadth.

Recently, we have described several approaches to reduce plasma anti-base antibody responses in NHPs.^20^ We have found that FP-carrier conjugate priming, either alone or in a cocktail with a SOSIP-stabilized trimer, can reduce anti-base responses compared to immunizing with trimers only. Furthermore, long immunization intervals can reduce base responses in NHPs primed with FP and subsequently boosted with trimers, and these reduced responses correlate with increased neutralization breadth. Although these findings provide insight into how the usage of FP-carrier conjugates can improve vaccine regimens, it has been unclear whether the reduced anti-base responses were due to the long FP immunization intervals, the long trimer-immunization intervals, or a combination of both. Also, whether other immunization regimen parameters, such as the adjuvant, could reduce anti-base responses has yet to be explored.

Here, we analyze prime-boost immunization regimens of FP-carrier conjugates and soluble SOSIP-stabilized trimers in NHPs to identify optimal intervals and adjuvants for reducing anti-base antibody responses. As the BG505 DS-SOSIP trimer is being tested in clinical trials, we focus on this trimer and use the same dose as reported in previous NHP studies.

## Results

### A long trimer interval, but not a long FP interval, reduces the anti-base antibody response in FP-primed NHPs

**Figure 1:**
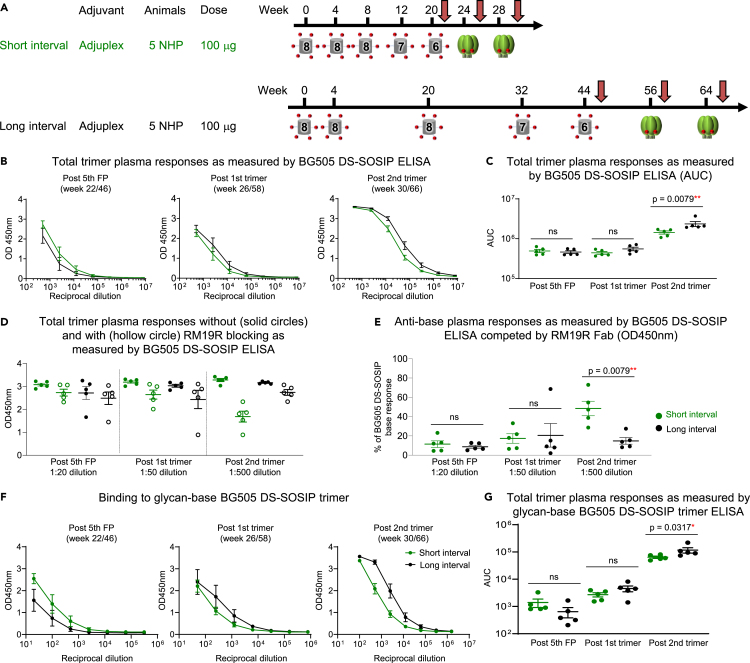
In FP primed/trimer boosted NHPs, long trimer interval, but not long FP prime interval, significantly impacts plasma anti-base responses For all regimens, NHP plasma at three key time points of two weeks after the fifth FP immunization, the first trimer immunization and the second trimer immunization were analyzed (indicated with a red arrow). Two groups of five animals each were first primed three times with FP8-KLH followed by FP7-KLH, FP6-KLH, and two BG505 DS-SOSIP immunizations, with Adjuplex and I.M. Green and black circles and lines represent NHPs from short interval groups and long-interval groups, respectively. (A) One group of five animals were immunized with short intervals at weeks 0, 4, 8, 12, 20, 24, and 28, and one group of five animals were immunized with long intervals at weeks 0, 4, 20, 32, 44, 56, and 64. (B) Plasma binding to BG505 DS-SOSIP by ELISA as measured by OD450nm plotted against plasma dilution factors for short and long interval groups at the three key time points of two weeks post fifth FP (left panel), two weeks post first trimer (second panel from left) and two weeks post second trimer (right panel). (C) Total trimer response as measured by ELISA BG505 DS-SOSIP AUC from the values in B. (D) Comparison of the total ELISA OD450nm values for binding to BG505 DS-SOSIP with (hollow circles) and without (solid circles) competition with RM19R Fab between the short and long interval groups at dilutions of 1:20, 1:50, and 1:500 for the three time points, respectively. (E) Percentage of response targeting the base region calculated from the values in D. The percentage was calculated by taking the difference between OD450 values without and with RM19R Fab, divided by the OD450 values without RM19R Fab blocking, and multiplied by 100. (F) Plasma binding by ELISA to glycan-base BG505 DS-SOSIP trimer, which has glycans added on the base region. (G) Trimer response as measured by AUC from the values in F. Two-tailed Mann-Whitney nonparametric tests were used for statistical analysis to assess p values for mean ± SEM. ∗: p < 0.05; ∗∗: p < 0.01; ∗∗∗: p < 0.001; ∗∗∗∗: p < 0.0001; ns, not significant. The data in C represent two independent experiments with similar results. See also Figure S1.

To investigate the effect of immunization intervals on anti-base antibody responses, we analyzed 10 NHPs that received five FP-KLH immunizations followed by two BG505 DS-SOSIP trimer boosts as previously described.^20^ Animals were divided into two groups that differed in their immunization intervals. The long interval group received five FP immunizations at weeks 0, 4, 20, 32, and 44 and two trimer immunizations at weeks 56 and 64, whereas the short interval group received five FP immunizations at weeks 0, 4, 8, 12, and 20 and two trimer immunizations at weeks 24 and 28 (Figure 1A). In a prior study,^20^ we analyzed animals in these two groups for total trimer responses and anti-base responses after the final trimer immunization. Here, we analyzed earlier time points to determine whether reduced base responses were due to the longer FP intervals, the longer trimer intervals, or a combination of both. We studied serum collected two weeks after the fifth FP immunization and two weeks after the first trimer immunization to compare with antibodies present two weeks post two trimer immunizations. The analysis of total trimer responses to BG505 DS-SOSIP by ELISA revealed no differences between short- and long-interval groups after the fifth FP and the first trimer immunization time points and a slightly (1.6-fold) higher binding response for animals in the long-interval group two weeks after the final trimer immunization (Figures 1B and 1C). Next, we carried out a competition-based ELISA using the base-specific RM19R Fab to quantify the percentage of antibody response targeting the base region as previously described.^20^ Previously, we demonstrated that anti-base antibody responses can be quantified similarly using either area under the curve (AUC) values at serial dilutions or OD450nm values at a single dilution when the total trimer ELISA responses are comparable. Therefore, in this study, we quantified the percentage of base responses for three time points by OD450nm values at a single dilution. Specifically, we chose the dilutions of 1:20, 1:50, and 1:500 for the time points at post fifth FP, post first trimer, and post second trimer immunizations, respectively, as these dilutions produced comparable OD450nm values for the total trimer response (Figure 1D). At the post fifth FP and post first trimer time points, we observed similarly low levels of anti-base responses for both groups. After the second trimer immunization, the short interval group was characterized by a significantly higher anti-base response, as previously described (Figure 1E).^20^ While the long interval group maintained an anti-base response at low levels, the percentage of response targeting the base region was increased in animals of the short interval group (Figure 1E). Overall, these data suggest that it is the long trimer interval, but not the long FP interval, that has an impact on the anti-base antibody response elicited after trimer immunization.

### Plasma non-base response based on glycan-base trimer binding correlates with FP response and neutralizing breadth in FP primed NHPs

To confirm the anti-base antibody responses analyzed via competition ELISA with the RM19R Fab, we performed ELISA using a glycan-base BG505 DS-SOSIP trimer^21^ for the three time points mentioned previously and quantitated the binding via AUC. This glycan-base trimer has three glycans per protomer added to the base region of BG505 DS-SOSIP at the amino-terminus (N-terminus) of gp120 and carboxy-terminus (C-terminus) of gp41, which significantly reduces the binding of base-specific monoclonal antibodies while maintaining the presentation of critical epitopes on CD4bs, V1V2, FP, and V3-glycan (Figures S1A and S1B). Similar to observations with the Fab competition experiments, there was no significant difference in binding to the glycan-base trimer between the two groups at two weeks post fifth FP and post first trimer immunizations. At two weeks post the second trimer immunization, higher binding responses to the glycan-base trimer were observed in the long interval group (Figures 1F and 1G). This further confirms that the anti-base antibody response is more strongly influenced by the interval length between trimer immunizations than that between the FP immunizations.

**Figure 2:**
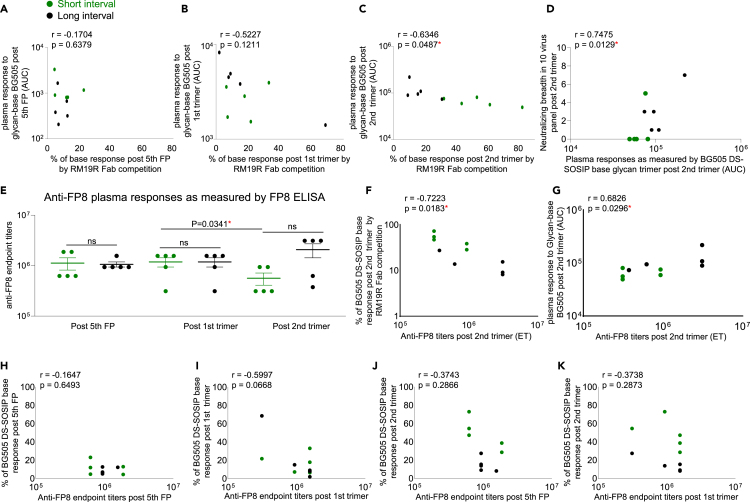
In FP primed/trimer boosted NHPs, long trimer interval after FP prime enhances FP responses and neutralizing breadth Green circles represent NHPs from short-interval group, and black circles represent NHPs from long-interval group. (A – C) Correlation analysis of the percentage of anti-base response based on RM19R Fab competition assay and non-base trimer response detected with glycan-base BG505 DS-SOSIP at the three key time points of two weeks post fifth FP immunization (A), two weeks post first trimer immunization (B) and two weeks post second trimer immunization (C). (D) Correlation of the AUC values of non-base trimer response detected with glycan-base BG505 DS-SOSIP and neutralizing breadth at two weeks post second trimer immunization. Neutralizing breadth is the number of neutralized wild-type viruses within a 10-virus panel in which all viruses contain the same FP8 sequence and are sensitive to FP-specific antibodies. (E) Anti-FP plasma response at the three key time points detected with ELISA assay as shown with mean ± SEM. Two-tailed paired t test was performed for post first and second trimer immunization for short-interval group. Two-tailed Mann-Whitney nonparametric tests were used for statistical analysis to assess p values for short and long interval groups at each time points. (F) Correlation of anti-FP8 ELISA endpoint titers obtained two weeks post second trimer immunization and the percentage of base response on BG505 DS-SOSIP at two weeks post second trimer immunization by RM19R Fab competition. ET stands for endpoint titer. (G) Correlation of anti-FP8 ELISA endpoint titers obtained two weeks post second trimer immunization and the AUC values of non-base trimer response detected with glycan-base BG505 DS-SOSIP at two weeks post second trimer immunization. ET stands for endpoint titer. (H and I) Correlation between Anti-FP8 ELISA endpoint titers and the percentage of base response as determined by BG505 DS-SOSIP competition ELISA with RM19R Fab two weeks post fifth FP immunization (H) and two weeks post first trimer immunization (I). (J and K) Correlation between anti-FP8 ELISA endpoint titers two weeks post fifth FP immunization (J), two weeks post first trimer immunization (K) and the percentage of base response two weeks post second trimer immunization, as determined by BG505 DS-SOSIP competition ELISA with RM19R Fab. Two-tailed Pearson correlation coefficient test was performed for correlation analysis. ∗: p < 0.05; ∗∗: p < 0.01.

To validate the use of glycan-base trimers as a means of analyzing non-base trimer responses, we compared the binding response to the glycan-base trimer with the percentage of anti-base response calculated from RM19R Fab competition. No correlation was detected between these two analysis methods for samples collected two weeks post the fifth FP and two weeks post the first trimer (Figures 2A and 2B). However, at two weeks post the second trimer, there was a strong negative correlation (r = −0.6346, p = 0.0487), indicating that the results from these two methods were consistent with each other (Figure 2C).

Furthermore, we observed a positive correlation between glycan-base trimer binding and neutralization breadth on a prior established panel of 10 Tier-1 and Tier-2 FP-sensitive strains at two weeks post two trimer immunizations (r = 0.7475, p = 0.0129) (Figure 2D).^8^^,^^10^^,^^11^^,^^20^ Together with our previous finding that percentages of anti-base response within total trimer response inversely correlate with neutralizing breadth on the 10 FP-sensitive panel,^20^ the data indicate that non-base responses correlate with neutralization breadth and confirm our previous findings.^20^

To further analyze plasma FP responses for the three key time points, we performed plasma ELISA with biotinylated FP comprising the most prevalent eight amino acid sequence (FP8_v1).^22^ While there was no significant difference in anti-FP titers between the two groups at any time point, the titers for the long interval group trended higher at two weeks post two trimer immunizations, and anti-FP responses were reduced after the second trimer immunization for the short trimer interval group, but not for the long interval group (Figure 2E), indicating that the short trimer interval may be disrupting epitope-specific antibodies from developing and expanding (Figure 2E). Statistical analysis revealed that, at two weeks post the second trimer, anti-FP titers were inversely correlated with the percentage of base response as determined by RM19R competition (Figure 2F) and positively correlated with binding to the glycan-base trimer (Figure 2G). Anti-FP titers post fifth FP immunization and post first trimer immunization were not correlated with base responses at any of these time points (Figures 2H–2K), further confirming that a longer FP interval does not predict nor impact the anti-base antibody responses elicited after trimer immunization.

### Long immunization interval reduces anti-base response in trimer-only immunized NHPs

**Figure 3:**
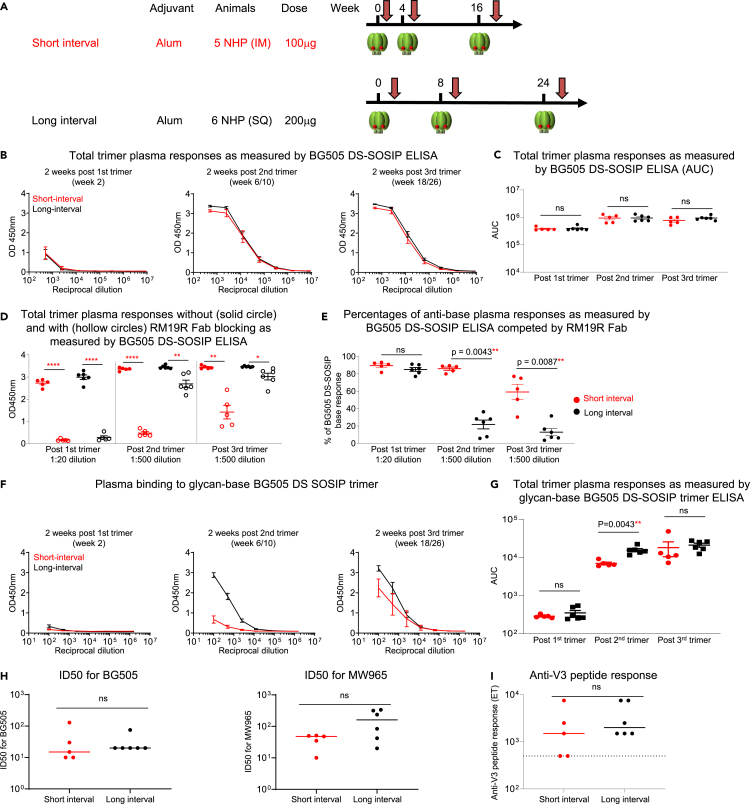
Long trimer interval reduces plasma anti-base responses in trimer-only immunized NHPs but does not enhance neutralizing activity For all regimens, NHP plasma at three key time points of two weeks after the first, second, and third trimer immunizations were analyzed. Red circles and lines represent NHPs from short-interval group, and black circles and lines represent NHPs from long-interval group. (A) Immunization regimen of short-interval and long-interval NHPs in trimer only immunization. Six NHPs were immunized with BG505 DS-SOSIP at weeks 0, 8, and 24 with S.Q (long interval), while five NHPs were immunized with BG505 DS-SOSIP at weeks 0, 4, and 16 with I.M. (short-interval). Both groups used Alum as adjuvant. (B) Plasma response to BG505 DS-SOSIP trimer at three key time points. (C) Total trimer response as measured by ELISA BG505 DS-SOSIP AUC values. (D) Comparison of the total ELISA OD450nm values for binding to BG505 DS-SOSIP with (hollow circles) and without (solid circles) competition with RM19R Fab between the short and long interval groups at dilutions of 1:20, 1:500, and 1:500 for the three time points, respectively. Two-tailed paired-t test were used for statistical analysis to assess p values with and without Fab competition, with mean ± SEM. (E) Percentage of trimer response targeting the base region calculated from the values in D. The percentage was calculated by taking the difference between OD450 values without and with RM19R Fab, divided by the OD450 values without RM19R Fab blocking, and multiplied by 100. (F) Plasma binding to glycan-base BG505 DS-SOSIP trimer, which has glycans added on the base region. (G) Plasma responses as measured by glycan-base BG505 DS-SOSIP trimer AUC from F. (H) Neutralizing ID50 on BG505 and MW965 at two weeks post third trimer immunization. (I) Anti-V3 peptide response at two weeks post third trimer immunization. Two-tailed Mann-Whitney nonparametric tests were used for statistical analysis for D-I, to assess p values for mean ± SEM. ∗: p < 0.05; ∗∗: p < 0.01; ∗∗∗: p < 0.001; ∗∗∗∗: p < 0.0001; ns, not significant. The data in C represent two independent experiments with similar results. See also Figures S1–S3.

We next investigated whether a longer trimer interval would reduce base-directed responses in the absence of an FP prime. We selected 11 NHPs in two vaccine groups that received three BG505 DS-SOSIP trimer immunizations.^23^ The two vaccine groups differed in their immunization intervals, with the long interval group receiving immunizations at weeks 0, 8, and 24 and the short interval group receiving their immunizations at weeks 0, 4, and 16, in addition to different immunization route (S.C. vs. I.M.) and dose (200 μg vs. 100 μg) (Figure 3A). The short interval group had been previously analyzed for anti-base responses two weeks post two trimer immunizations,^20^ whereas the long interval group was from a new study. We analyzed plasma antibody responses for differences between the two groups at three time points comprising two weeks post each trimer immunization (weeks 2, 10, and 26 for the long interval group and weeks 2, 6, and 18 for the short interval group). We first analyzed total trimer response to BG505 DS-SOSIP via ELISA and found no significant difference between the two groups at any of the time points. This indicated that the dose and immunization route did not impact the total trimer response (Figures 3B and 3C). To compare the percentage of base responses across the three time points, we chose a starting dilution of 1:20 for the post one trimer time point and a dilution of 1:500 for the time points of post two and post three trimer immunizations, as these dilutions gave rise to comparable OD450nm values for the total trimer response across the three time points. We quantified the percentage of base-response by RM19R competition and found that while there was no significant difference in the percentage of anti-base antibody responses after the first trimer immunization, at two weeks post the second trimer immunization, the long interval group was characterized by a significantly lower percentage of anti-base antibody responses (average of 22% and 86% for the long and short interval groups, respectively), and this significant difference between the two groups was maintained post the third trimer immunization (average of 13% and 59% for the long and short interval groups, respectively) (Figures 3D and 3E). This substantial difference between the two groups demonstrated that the long interval between trimer immunizations in NHPs immunized with trimer only can significantly reduce the anti-base antibody responses.

To confirm the results obtained by RM19R competition that the long interval between immunizations reduced anti-base responses in NHPs immunized only with trimers, we performed ELISA with the glycan-base trimer at the same three time points. We observed the long interval group to have significantly higher binding to the glycan-base trimer two weeks post two trimer immunizations (Figures 3F and 3G). While there was no significant difference in binding at two weeks post the third trimer, the ELISA titers for the long interval group also trended higher, and we further explored how the anti-base antibody response developed and changed throughout the immunization time course. We included more time points and assayed plasma responses to the BG505 DS-SOSIP trimer and the glycan-base BG505 DS-SOSIP trimer. For the short interval group, the time points analyzed included weeks 2, 4, 6, 10, 14, and 18, and for the long interval group, the time points analyzed included weeks 2, 6, 8, 10, 14, 22, and 26 (Figure S2). With the addition of the new time points, our explorations revealed that, at the time for the second trimer immunization (week 8 for the long interval group and week 4 for the short interval group), the long interval group showed higher antibody responses targeting the glycan-base trimer and the difference between these two NHP groups increased after the second trimer immunization. Specifically, at week 10, the long-interval group showed significantly higher binding to the glycan-base trimer than the corresponding time point at week 6 for the short-interval group. This suggests that the reduction in anti-base response post two trimers for the long interval group may be due to non-base-specific antibodies being developed between the first and second trimer immunizations. We note that post two trimer immunizations, the level of anti-base antibody responses for the long interval group was mostly maintained until it was further reduced post third trimer immunization (Figure S2B). Furthermore, for the short interval group, the high anti-base antibody response was maintained after the second trimer immunization from week 6 to week 14. Although the anti-base response was reduced after the third trimer immunization, this response did not reach as low as that elicited by the long interval group. We thus conclude that the initial interval length between the first and second trimer is most critical for reducing the anti-base antibody response and for eliciting a greater proportion of antibodies targeting other epitopes on the trimer. However, since these two groups also differed in their dosage and immunization route, we could not fully exclude the possibility that S.C. immunization and higher dosage contributed to the reduced anti-base response in the long-interval group. Interestingly, we found that plasma neutralizing inhibitory dilution (ID50) titers post three trimer immunizations were similar between these two groups as measured on strains BG505 and MW965 (Figure 3H), with similar plasma anti-V3 peptide ELISA titers (Figure 3I), despite different anti-base responses. In addition, no correlation was observed between anti-base response and ID50 titers (Figures S3A–S3E).

### FP prime and trimer boost immunizations elicit antibodies with higher avidity to trimer compared to trimer-only immunizations in NHPs

**Figure 4:**
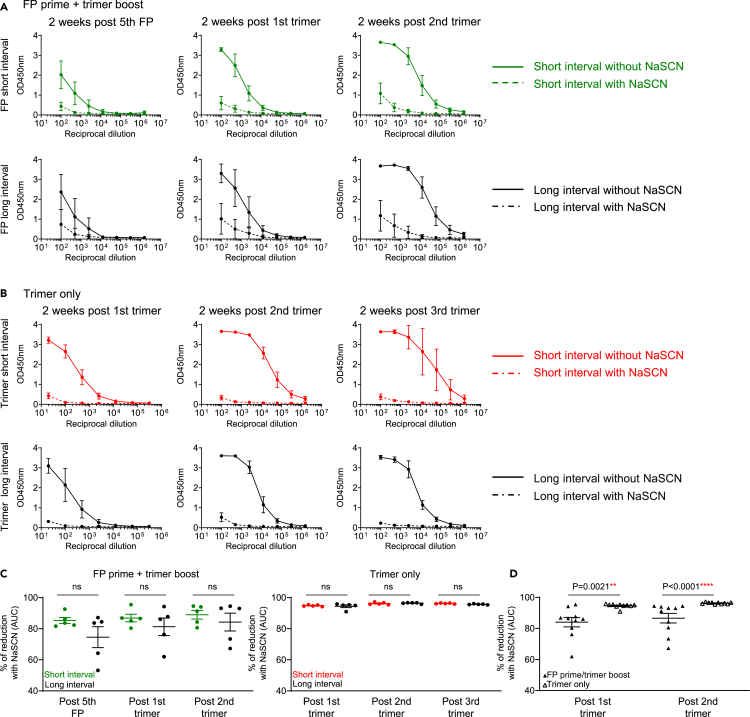
Differential binding to BG505 DS-SOSIP with and without NaSCN shows that NHPs immunized with FP prime followed by trimer boost elicit antibodies with higher avidity compared to trimer-only immunized NHPs (A and B) Plasma binding to BG505 DS-SOSIP without (solid line) or with NaSCN (dashed line) by ELISA as measured by OD450nm for each group are plotted against plasma dilution factors for FP prime+trimer boost group (A) and trimer only immunized group (B) at the three key time points of two weeks post fifth FP immunization (left panel), two weeks post first trimer (second panel from left) and two weeks post second trimer (right panel) for (A); two weeks post first trimer (left panel), two weeks post second trimer (second panel from left) and two weeks post third trimer (right panel) for (B). (C) Percentage of plasma binding reduction with NaSCN, calculated with AUC values from A and B for FP primer/trimer boost NHPs (left) and trimer only NHPs (right). (D) Percentage of plasma binding reduction in FP prime/trimer boost NHPs (solid triangle) and trimer only NHPs (empty triangle). FP prime/trimer boost with short or long intervals are combined as “FP prime/trimer boost” group, and trimer only groups with short or long intervals are combined as “trimer only” group. The reduction was calculated by taking the difference between AUC values without and with NaSCN divided by the AUC values without NaSCN and multiplied by 100. Two-tailed Mann-Whitney nonparametric tests were used for statistical analysis to assess p values for mean ± SEM. ∗: p < 0.05; ∗∗: p < 0.01; ∗∗∗: p < 0.001; ∗∗∗∗: p < 0.0001; ns, not significant.

To explore if antibody avidity was impacting the anti-base response that we measured by Fab competition assay or by glycan-base trimer, we analyzed the avidity of antibody responses targeting BG505 DS-SOSIP by carrying out a differential ELISA in the presence or absence of sodium thiocyanate (NaSCN). The addition of NaSCN after plasma incubation disrupted binding and thus allowed us to differentiate between weak and high-avidity binding.^24^^,^^25^ No significant difference in avidity between the two FP-primed NHP groups (Figures 1A, 4A, and 4C) or trimer-primed NHP groups (Figures 3A, 4B, and 4C) was observed at any of the time points, indicating that avidity was not the cause of different anti-base responses between the two FP primed groups or between the two trimer primed groups. However, when comparing between the FP primed and trimer primed NHPs, at two weeks post the first trimer and two weeks post the second trimer, the FP primed NHPs showed significantly higher avidity to BG505 DS-SOSIP (Figures 4A, 4B, and 4D). As the plasma antibodies from FP-prime trimer-boost NHPS have increased specificity for FP, this could indicate that FP-specific antibodies have higher avidity to the trimer compared to the antibodies targeting base or other epitopes elicited by trimer-only immunized NHPS. Of note, the FP primed NHPs experienced more immunizations compared to trimer-only immunized NHPs, thus the higher avidity could also be due to longer maturation time for antibodies targeting FP epitopes. On the other hand, the long trimer interval reduced anti-base response in both FP prime/trimer boost and in trimer-only immunized NHPs, but the long trimer interval did not enhance serum antibody avidity in both groups, indicating the enhanced avidity to trimer was not solely due to the long immunization time in FP primed animals. Nevertheless, the enhanced serum antibody avidity to trimer in FP-primed NHPs may contribute to the enhanced neutralizing breadth and titers.

### Memory B cell analysis confirms anti-base responses observed in plasma

**Figure 5:**
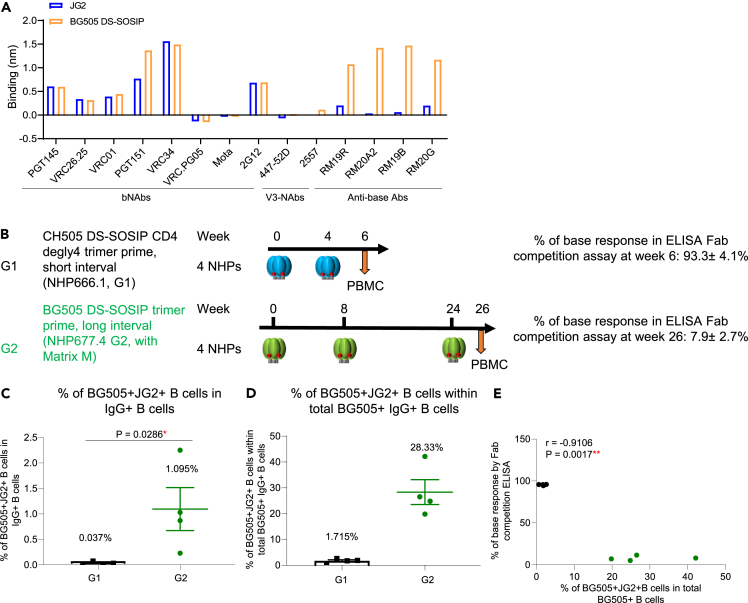
Frequency of non-base binding trimer-specific IgG+ B cells inversely correlates with the prevalence of anti-base responses detected in plasma (A) JG2 trimer showed similar binding to broadly neutralizing antibody panels and V3-panel, while significantly reduced binding to the anti-base monoclonal antibodies, detected with Octet. (B) Immunization schema of NHP study G1 (black), with CH505 DS-SOSIP trimer primed at weeks 0 and 4, and blood drawn at week 6, and Immunization schema of NHP study G2 (green), with BG505 DS-SOSIP trimer primed at weeks 0, 8, and 24, and blood drawn at week 26. G1 and G2 showed 93% and 8% anti-base response within total trimer response, respectively, detected with ELISA RM19R Fab competition assay. FACS analysis was performed using BG505 DS-SOSIP and JG2 trimer probes for G1 and G2 PBMC. (C) Frequency of IgG+ B cells that bind both BG505 DS-SOSIP trimer and JG2 glycan-base trimer in IgG+ B cells. (D) Frequency of BG505+JG2+ B cells within BG505+ IgG + B cells. (E) Frequencies of BG505+JG2+ B cells correlate inversely with percentage of trimer-base response detected with ELISA RM19R Fab competition assay. Two-tailed Mann-Whitney nonparametric tests were used for statistical analysis to assess p values for mean ± SEM. ∗: p < 0.05; ∗∗: p < 0.01; ∗∗∗: p < 0.001; ∗∗∗∗: p < 0.0001; ns, not significant. Correlation analysis was performed using a two-tailed Pearson correlation coefficient test. See also Figure S4.

We next sought to investigate if antigen-specific memory B cells exhibited a similar pattern with regard to base-specificity as the plasma response. Because the glycan-base trimer BG505 DS-SOSIP failed to form a well-assembled prefusion conformation when an avi-tag was added in the C-terminal of gp41, we used a new glycan-base probe (BG505 JG2) for fluorescence-activated cell sorting (FACS). This glycan-base probe had two additional glycans per protomer at the base region to cover the artificial base of the soluble Env trimer.^26^ Prior to performing the FACS analysis, we confirmed that this JG2 probe showed similar binding to broadly neutralizing antibodies and eliminated binding to anti-base monoclonal antibodies via Biolayer Interferometry assay (Figure 5A). To compare the B cell responses with plasma antibody responses, we selected two groups (G1 and G2) of NHPs: Group G1 (four NHPs) were immunized twice at weeks 0, and 4 with a CH505 DS-SOSIP trimer that has four glycans removed around the CD4-binding site (CD4bs), and their plasma showed an average of over 90% anti-base response from total anti-BG505 trimer response at week 6; Group G2 (four NHPs) were immunized three times at weeks 0, 8, and 24 with BG505 DS-SOSIP, and their plasma showed an average of 7.9% anti-base response within total anti-BG505 trimer response at week 26 (Figure 5B). We performed FACS analysis using both the BG505 JG2 probe and BG505 DS-SOSIP probe to analyze the percentage of IgG+ B cells that were dual-specific to BG505 DS-SOSIP and BG505 JG2 trimer (glycan-base trimer). We then calculated the frequency of dual-specific B cells out of the total BG505 trimer positive B cells. This B cell analysis showed that NHPs from group G2 two weeks post three trimer immunizations had significantly higher frequency of BG505+JG2+ B cells in total IgG+ B cells than NHPs from group G1 two weeks post two trimer immunizations (1.095% vs. 0.037%, Figures 5C and S4A) and a higher frequency of BG505+JG2+ B cells within total BG505 + B cells (28.33% vs. 1.715%, Figure 5D). In addition, we observed a strong inverse correlation between BG505+JG2+ B cell frequency and the percentage of anti-base response in plasma at the same time points (r = −0.9106, p = 0.0017, Figure 5E). Together, these data indicate the B cell analysis to be consistent with the plasma antibody analysis and the frequency of non-base specific memory B cells to develop at a similar rate to the plasma non-base antibody response, although this does not necessarily imply an enhanced epitope-specific immune response targeting neutralizing epitopes, as here we did not specify the epitope specificity of the non-base antibody response.

### Adjuvants impact the epitope-specific response in trimer-only immunized NHPs

**Figure 6:**
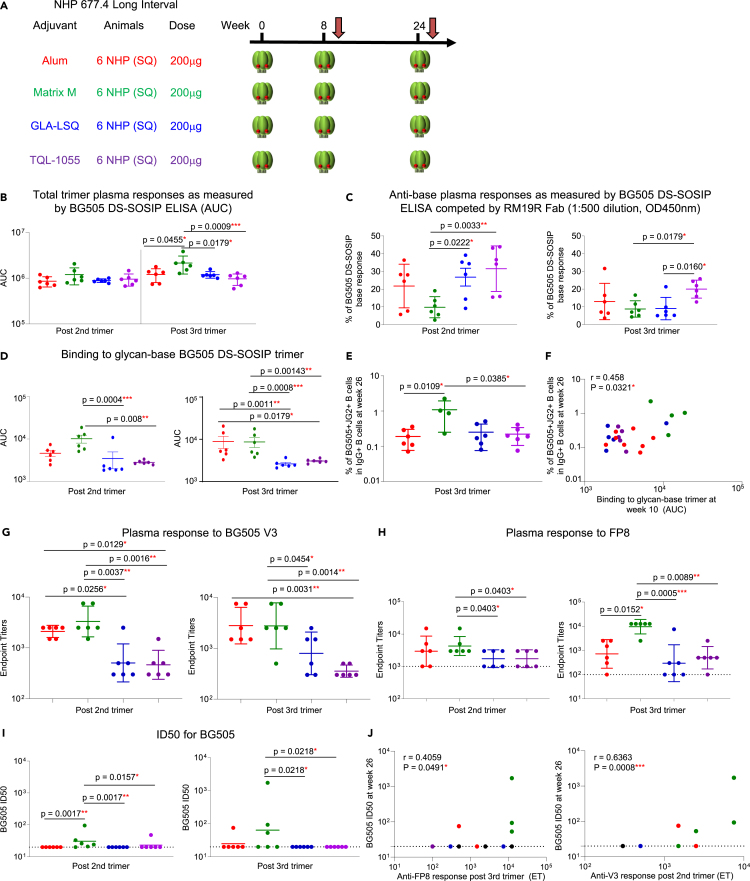
In long-interval trimer immunization, adjuvant impacts neutralizing titers and anti-base response For all regimens, NHP plasma at two key time points of two weeks after the 2nd and 3rd trimer immunizations were analyzed. Red, green, blue and purple circles represent NHPs with Alum, Matrix M, GLA-LSQ and TQL-1055, respectively. (A) Immunization regimen. Six NHPs per group were immunized with BG505 DS-SOSIP at weeks 0, 8, and 24, S.Q. with different adjuvants. (B) Plasma response to BG505 DS-SOSIP trimer at two weeks post 2nd trimer and 3rd trimer immunizations. (C) Anti-base plasma responses as measured by BG505 DS-SOSIP ELISA with RM19R Fab competition (1:500 dilution, OD450nm). (D) Total trimer response as measured by glycan-base BG505 DS-SOSIP trimer AUC values. (E) Percentages of IgG+ B cell that bind both BG505 DS-SOSIP trimer and JG2 trimer probes in PBMC after 3rd trimer immunization. (F) Percentages of BG505+JG2+ B cells correlate with plasma response to glycan-base BG505 trimer. (G and H) Plasma response against V3 peptide (G) and FP8 peptide (H) at 2 weeks post 2nd and 3rd trimer immunization. (I) ID50 titers on BG505 post 2nd and 3rd trimer immunization. (J) Plasma anti-FP (left) and V3 (right) responses correlate with BG505 ID50, with all data from different groups combined. Data are shown with mean ± SEM for multiple comparison analysis, with Kruskal-Wallis test followed with two-stage linear step-up procedure of Benjamini, Krieger and Yekutieli tests to assess p values between different adjuvant groups. ∗: p < 0.05; ∗∗: p < 0.01; ∗∗∗: p < 0.001; ∗∗∗∗: p < 0.0001; ns, not significant. The Data in B–F represent 2 independent experiments with similar results. Correlation analysis was performed using a two-tailed Pearson correlation coefficient test. See also and Figures S1, S4, S5, and S7.

Adjuvants play an important role in enhancing vaccine elicited immune responses.^27^ How different adjuvants given with soluble trimer immunization affects the elicitation of trimer-base response has been unknown. Here, we sought to investigate the impact of different adjuvants on the anti-base response and neutralization outcomes in NHPs immunized with BG505 DS-SOSIP. To do so, we analyzed several cohorts of NHPs receiving different adjuvants. In the first cohort, 24 NHPs in four groups were immunized with BG505 DS-SOSIP at weeks 0, 8, and 24 with either Alum, Matrix M, GLA-LSQ or TQL-1055 adjuvants. All NHPs were immunized with the same dosage (200 μg) and immunization route (S.C.) (Figure 6A). We analyzed plasma antibody responses to BG505 DS-SOSIP by ELISA in the presence or absence of RM19R Fab, and to the glycan-base BG505 DS-SOSIP trimer at the two time points of two weeks post two trimer immunizations (week 10) and two weeks post three trimer immunizations (week 26) (Figures 6B–6D). The four groups showed similar trimer responses two weeks post two trimer immunizations, but after three trimer immunizations, the Matrix M group showed the highest trimer response (Figure 6B). The percentage of anti-base response within the total trimer response was then calculated using the RM19R Fab competition assay. At a 1:500 dilution, the Matrix M group showed the lowest anti-base response two weeks post two trimer immunizations, and both the Matrix M and GLA-LSQ groups showed lower anti-base response than the TQL-1055 group at post three trimer immunizations (Figure 6C). We then tested non-base responses to the glycan-base BG505 DS-SOSIP trimer via ELISA. Consistent with Fab competition results in Figure 6C, the Matrix M group showed the highest response to the glycan-base trimer post two trimer immunizations, whereas at post three trimer immunizations, the Alum group showed increased levels of non-base response, and both Alum and Matrix M groups showed higher non-base response than the other two groups (Figure 6D).

To confirm the previous ELISA results on the impact of adjuvants, we measured the antigen-specific IgG+ B cell response with BG505 DS-SOSIP and BG505 JG2 (glycan-covered base) probes via FACS analysis (Figures 6E, S4A, and S4B). At two weeks post third trimer immunization, the Matrix M group showed a significantly higher frequency of BG505+JG2+ B cells than the other three groups, indicating that more B cells were targeting non-base trimer epitopes (Figure 6E). In addition, we found a correlation between non-base response (binding to glycan-base BG505 DS-SOSIP) at two weeks post second trimer immunization and BG505+JG2+ B cell frequency two weeks post third trimer immunization (Figure 6F), indicating that early non-base plasma responses may be associated with later development of non-base targeting memory B cell responses.

After discovering similar patterns for both plasma and B cell responses, we wanted to investigate which epitopes are contributing to the difference of non-base responses. We tested plasma responses to the BG505 V3 peptide (Figure 6G) and FP (Figure 6H) via ELISA and found that the Alum and Matrix M groups showed higher response to the V3 peptide than the other two groups at two weeks post second and third trimer immunizations (Figure 6G). Plasma binding to FP was similar for Alum and Matrix M groups, with both showing higher FP-responses than the other two groups post two trimer immunizations, but post three trimer immunizations, the Matrix M group showed the highest anti-FP response (Figure 6H). We then compared the plasma neutralizing activity, and the Matrix M group showed the highest ID50 titers on autologous BG505 at both post two and three trimer immunizations, although the ID50 values were still quite minimal for this group (Figure 6I), and the Alum and Matrix M groups showed higher MW965 ID50 titers than the other two groups (Figure S5A). Correlation analyses were then performed between previous tested parameters, and we found positive correlations between anti-FP and anti-V3 responses to BG505 ID50 titers (Figure 6J). The Matrix M group showed the highest ID50 titers on Tier 1a MW965 (Figure S5A) and a positive correlation was found between plasma anti-BG505 V3 peptide responses and the MW965 neutralization ID50 titers at both two weeks post two and three trimer immunizations (Figure S5B). The anti-BG505 V3 peptide and anti-BG505 FP responses after two trimer immunizations were also negatively correlated with the percentage of anti-base response at the same time point, indicating that the development of both V3 and FP responses may be responsible for driving the low anti-base response seen in these groups (Figure S5C). To elucidate if differences between different adjuvant groups were due to different activation statuses of germinal centers, levels of CXCL13, a plasma biomarker of germinal center activation, were measured.^28^ Plasma CXCL13 concentrations were similar between all four groups after one and two trimer immunizations, indicating the activation of the germinal centers may be similar between the four different adjuvanted NHP groups (Figure S5E). To assess the impact of adjuvants on the accessibility of trimer epitopes, a Meso Scale Discovery (MSD) assay was performed for BG505 DS-SOSIP trimer mixed with different adjuvants (Figure S5F). BG505 DS-SOSIP trimer, in the presence or absence of adjuvant, showed strong binding to VRC01, a CD4bs-directed antibody, and to PGT145, a V2 apex-directed antibody, while little binding to the negative control CR9114 antibody was observed. Alum mixed with trimer was the only adjuvant which appeared to have lower binding to open trimer-specific antibodies F105, 447-52D, and 3074 (Figure S5F).

**Figure 7:**
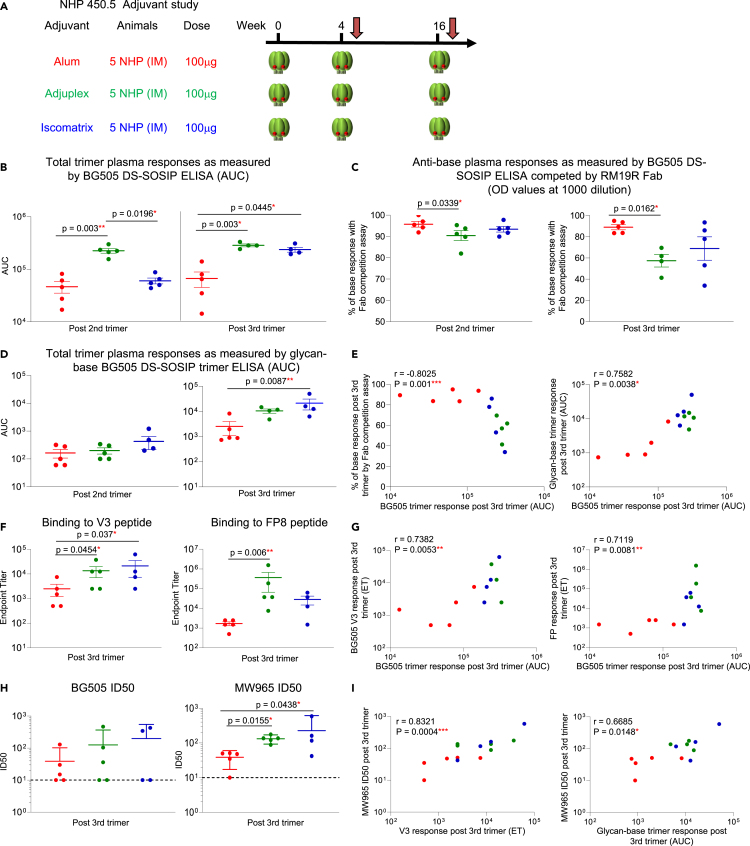
In short-interval trimer immunization, adjuvant impacts total trimer response and anti-base response For all regimens, NHP plasma at two key timepoints of two weeks after the second and third trimer immunization were analyzed. Red, green and blue circles represent Alum, Adjuplex and Iscomatrix groups, respectively. (A) Immunization regimen. Five NHPs per group were immunized with BG505 DS-SOSIP trimer at weeks 0, 4, and 16, I.M. with different adjuvants. (B) Total trimer response as measured by ELISA BG505 DS-SOSIP AUC values. (C) Percentage of anti-base response measured via competition with RM19R Fab. (D) Trimer response as measured by glycan-base BG505 DS-SODIP. (E) Correlation between total trimer response and anti-base response, calculated using ELISA assay with RM19R Fab competition assay (percentages, left) or glycan-base trimer (right) in ELISA assay. (F) Plasma response to BG505 V3 peptide (left) and FP peptide (right) as measured by ELISA two weeks post third trimer immunization. (G) Correlation between total trimer response vs. anti-V3 (left) and anti-FP (right) response measured by ELISA two weeks post third trimer. (H) ID50 neutralization titers against BG505 (left) and MW965 (right) two weeks post third trimer immunization. Dotted lines represent the starting dilution at 1:10. (I) Correlation between MW965 ID50 neutralization titers and anti-V3 peptide response (left) and glycan-base BG505 DS-SOSIP trimer response (right) measured via ELISA. Data are shown with mean ± SEM for multiple comparison analysis, with Kruskal-Wallis test followed with two-stage linear step-up procedure of Benjamini, Krieger and Yekutieli tests to assess p values between different adjuvant groups. ∗: p < 0.05; ∗∗: p < 0.01; ∗∗∗: p < 0.001; ∗∗∗∗: p < 0.0001; ns, not significant. The data in B–F represent 2 independent experiments with similar results. Correlation analysis was performed using a two-tailed Spearman correlation coefficient test. See also Figures S1, S6, and S7.

As we found that adjuvants play a role in anti-base antibody responses in long trimer interval NHPs, we also wanted to see if the same held true for short trimer interval NHPs. We immunized a second cohort comprising three groups of NHPs with BG505 DS-SOSIP mixed with either Alum, Adjuplex, or Iscomatrix at weeks 0, 4, and 16 (Figure 7A). We analyzed plasma antibody responses to BG505 DS-SOSIP, with and without RM19R Fab competition, and to the glycan-base BG505 DS-SOSIP trimer at two weeks post the second and third trimer immunizations (weeks 6 and 18, respectively) (Figures 7B–7D). At two weeks post two trimer immunizations, the Adjuplex group showed the highest plasma response to the trimer, and post three trimer immunizations, both Adjuplex and Iscomatrix groups showed similar trimer responses that were significantly higher than the Alum group (Figures 7B, S6A, and S6B). We then calculated the percentage of base response within the total trimer response by RM19R Fab competition assay. At a 1:1000 dilution, all three groups showed dominant (>90%) anti-base responses after the second trimer immunization (Figure 7C, left panel). After the third trimer immunization, the anti-base responses decreased in Adjuplex and Isocomatrix groups, but not in the Alum group (Figure 7C right panel; Figures S6A and S6B). The Adjuplex group showed the lowest anti-base response two weeks post the second and third trimer immunizations, with significantly lower percentage of anti-base response than the Alum group post second and third trimer immunization (Figure 7C). We then tested anti-base responses with the glycan-base BG505 DS-SOSIP trimer via ELISA, and the Alum group showed the lowest response post second and third trimer immunizations, with both Adjuplex and Iscomatrix groups showing higher responses than the Alum group post third trimer immunization (Figure 7D). As the Alum group elicited the lowest overall trimer response, we wanted to determine whether this could be contributing to the lower response with the glycan-base trimer and Fab competition assay. To do so, we performed correlation analysis and indeed found strong correlations between trimer response and anti-base response detected with either Fab competition or with glycan-base trimer (Figure 7E). To map the epitopes for the elicited plasma antibody responses, we performed ELISA for the plasma binding to the BG505 V3 peptide and FP (Figure 7F). The Adjuplex group showed higher V3 and FP responses than the Alum group at both time points, and both V3 and FP responses were correlative with total trimer response (Figure 7G). For plasma neutralization activity, Adjuplex and Iscomatrix groups showed higher ID50 titers on homologous BG505 and Tier 1 MW965 virus than the Alum group after three trimer immunizations (Figure 7H). We did not find any correlation between anti-base response, anti-FP or anti-V3 response to BG505 ID50 neutralizing titers (Figures S6C–S6F), but found correlations between V3 and glycan-base trimer responses to MW965 ID50 (Figure 7I). CXCL13 concentration was similar between the three groups after the first trimer immunization, indicating the activation of germinal center responses may be similar (Figure S6G). The MSD assay of trimer mixed with different adjuvants showed similar binding responses for VRC01 and PGT145, whereas Adjuplex and Iscomatrix mixed with trimer showed higher binding to V3-specific antibodies F105, 447-52D compared to Alum (Figure S6H).

As immunization with BG505 DS-SOSIP trimer adjuvanted with Matrix M and Adjuplex showed enhanced FP response and reduced anti-base response in NHPs, we further tested the antigenicity of trimer mixed with Alum, Adjuplex, or Matrix M with anti-FP antibody VRC34.01, anti-gp120/gp41 interface antibodies PGT151, 35022, 8ANC195, and anti-base antibodies RM19R, RM20A2, RM19B1, and RM20G in MSD assay (Figure S7). None of the tested adjuvants changed the epitope accessibility on FP, gp120/gp41 interface (Figure S7A) or on the base region (Figure S7B), as similar binding responses were detected for trimer alone or for trimer mixed with different adjuvants.

## Discussion

The elicitation of protective neutralizing antibodies that target conserved Env-epitopes is critical for an effective HIV-1 vaccine. While advances in vaccine design have led to the production of SOSIP-stabilized Env trimers as candidate vaccines, these trimers are suboptimal due to their inclusion of a highly immunogenic glycan-free base that is absent on native virions. Additionally, efforts to engineer Env trimers with the base region masked with added glycans have failed to result in enhanced neutralization titers or breadth due to the dominant neo-epitopes at the base region.^21^ As such, identifying ways to both reduce anti-base antibody responses and improve epitope-specific antibodies could lead to improved vaccine outcomes. We have previously quantified anti-trimer base response within total trimer response in NHPs primed with SOSIP-stabilized soluble trimers and FP conjugates and found that immunization with trimer alone leads to more than 90% of the total trimer response targeting the base region, while priming with FP alone or in a cocktail with trimer significantly reduces the anti-base response.^20^ In addition, the FP-prime/trimer-boost regimens with long intervals elicited reduced anti-base responses and improved neutralization breadth. Here, to expand upon these findings, we utilized glycan-base trimers to detect the plasma antibody responses and memory B cell responses to non-base trimer regions in NHPs immunized with FP-carrier conjugates and boosted with SOSIP trimers. We found that long intervals for trimer immunizations, but not long intervals for FP immunizations, reduced the anti-base response, and the reduced anti-base response was associated with higher anti-FP response and neutralizing breadth for FP primed NHPs. This further confirms our previous finding that the development of FP-specific antibody responses lead to the lower anti-base response and enhances heterologous neutralizing activity.^20^

Furthermore, we looked at the impact of interval length in trimer only immunized NHPs and found that the long interval between the first and second trimer immunizations played a major role in reducing the anti-base response. This finding provides another vaccine design strategy to decrease antibody responses targeting the base region of SOSIP trimers. Previous studies have indicated that germinal center activity may be more robust with increased immunization interval length, with enduring affinity maturation and clonal migration.^29^^,^^30^^,^^31^ Thus, we hypothesize that the long interval between the first and second trimer may promote the development of diverse, non-base epitope-specific B cell responses, although we did not analyze lymph nodes in this study. While we did note differences in the immunization dosage and route between these two groups, their similar total trimer responses across all key time points indicates that these factors likely did not play a major role in eliciting total trimer response. Nevertheless, it is possible that these factors are also contributing to the difference in anti-base response seen between these two groups. Further exploration will be warranted to definitively rule out these potentially contributing factors.

In addition, although the anti-base response was significantly reduced in the long interval group, this reduction did not enhance neutralization activity and breadth at the analyzed time point. While it is possible that additional trimer immunizations could lead to higher neutralization activity, this likely indicates that although reduced anti-base antibody responses may play a role in the enhancement of Env-specific responses, the neutralization activity may be impacted by other factors that must also be optimized for improved neutralizing outcomes. One of the factors may be the avidity of the elicited antibodies. We found that the FP-primed groups showed higher avidity than trimer-primed groups. Since FP-specific antibodies were elicited in FP-primed NHPs, while anti-base antibodies dominated in trimer-primed NHPs, this suggests that the FP-specific antibodies may have higher avidity to the BG505 DS-SOSIP trimer than the anti-base antibodies. For the long interval trimer-only groups, it is possible that although the anti-base response is reduced, the response targeting other epitopes is still suboptimal in avidity and specificity, hindering the development of neutralizing responses. On the other hand, when FP-carrier conjugates were used to prime the immune response, the FP-specific antibodies developed with stronger avidity and further matured with subsequent trimer boosts, resulting in improved neutralization activity.

We then looked at the immunogen-specific IgG+ B cells with wild type and base-covered trimer probes and found the non-base specific memory B cell profile is consistent with the frequency of the plasma non-base response we calculated using an ELISA. This follows our hypothesis that the longer interval between trimer immunizations may allow for a more robust maturation process from the antigen-specific B cells. In addition to the impact of intervals, we also looked at the anti-base response in two new studies with seven groups of NHPs immunized with different adjuvants. We found that for the NHPs in the long interval groups, the adjuvant Matrix M group showed the least anti-base response, the best response to FP and V3 peptides, and the best neutralizing response. For the NHPs in the short interval groups, when compared to the Alum group, the Adjuplex and Iscomatrix groups showed higher trimer responses, lower anti-base responses, and higher responses to FP and V3 peptides, which is associated with higher neutralizing titers. Together, these two studies indicate that in addition to interval length, adjuvants can play a role in shaping the vaccine-induced total antibody response and epitope-specific antibody response. Of note, we saw the impact of interval length in these trimer-only immunized animals with different adjuvants, as anti-base responses are all below 50% of total trimer response in the long interval groups, while anti-base responses are all around 90% in the short interval groups after two trimer immunizations, regardless of the adjuvant used. Following a long interval between the second and third trimer immunization, all but Alum adjuvanted NHPs in the short interval groups showed significantly decreased anti-base response, suggesting that the long trimer interval helps to reduce the previously elicited dominant anti-base response. In addition, we detected a correlation between plasma anti-V3 responses with autologous neutralizing titers in the adjuvant comparison studies, which indicates that anti-V3 specific neutralizing antibodies were likely elicited in these animals. Since the plasma of trimer only immunized animals did not show neutralizing activity on heterologous Tier 2 viruses, these anti-V3 antibodies are likely strain-specific. As we did not look at immune responses in lymph nodes, a more in-depth analysis of the mechanisms behind how the adjuvant leads to different antibody responses is needed to fully understand this phenomenon.

Overall, our study indicates that FP priming, followed by a long interval trimer boost, in combination with an optimal adjuvant can enhance antibody responses targeting non-base neutralizing epitopes of the Env trimer, which may lead to better immunization outcomes. We also demonstrated the utility of base-covered trimers in quantifying the non-base trimer responses via ELISA and FACS analyses. Lastly, although reduced anti-base responses alone may not be sufficient for elicitation of potent HIV neutralizing activity, we believe it is likely a key component for consideration in the design and evaluation of HIV-1 soluble trimer vaccine candidates. FP priming, longer trimer immunization interval and optimized adjuvant assisted in increased antibody responses targeting non-base epitopes, and more immunizations with trimer to mature such non-base responses may lead to broadly neutralizing antibodies later on, as we only analyzed NHPs immunized with up to three trimer immunizations in this study. Indeed, broadly neutralizing antibodies isolated from NHPs with FP prime/trimer boost after more trimer immunizations showed potent and broad neutralizing activity^10^ and isolated monoclonal antibodies could protect NHPs from simian-human immunodeficiency virus (SHIV) infection (unpublished data). Therefore, we believe that reducing anti-base responses through means of increased trimer interval, optimized adjuvant, and epitope-targeting via prime methods may lead to enhanced vaccine outcomes. Indeed, recently reported Env-trimer immunization of NHPs^31^ with further engineered envelope trimer, escalating prime, saponin/monophosphoryl lipid A nanoparticles (SMNP) adjuvant, and long trimer immunization interval resulted in substantially more robust neutralizing responses; it will be interesting to combine escalating prime with the strategies revealed here for reducing base-directed responses and further improving the vaccine outcome.

### Limitations of the study

Our analyses of epitope-specific immune responses in the plasma and peripheral blood mononuclear cells (PBMC) are only from NHPs immunized with BG505 and CH505 DS-SOSIP trimers, so it is possible that immunization with trimers with different FP peptides/base region sequences or accessibility may yield different results. Also, our findings could be further confirmed with epitope-specific monoclonal antibody isolation and characterization, with different sets of probes targeting different epitopes. We also note that our analyses of epitope-specific immune responses can be achieved with other approaches, such as electron microscopy-based polyclonal epitope mapping (EMPEM) and draining lymph node germinal center B cell and Tfh cell analysis, which have been powerful tools to delineate the immune response to vaccine immunizations in animals and humans. Such approaches should also shed light on the mechanism of the antigen-specific B cell and T cell activation and maturation and provide clues for vaccine development and evaluation.

## Consortia

The VRC Production Program includes Nadia Amharref, Nathan Barefoot, Christopher Barry, Elizabeth Carey, Ria Caringal, Kevin Carlton, Naga Chalamalsetty, Adam Charlton, Rajoshi Chaudhuri, Mingzhong Chen, Peifeng Chen, Nicole Cibelli, Jonathan W. Cooper, Hussain Dahodwala, Marianna Fleischman, Julia C. Frederick, Haley Fuller, Jason Gall, Isaac Godfroy, Daniel Gowetski, Krishana Gulla, Vera Ivleva, Lisa Kueltzo, Q. Paula Lei, Yile Li, Venkata Mangalampalli, Sarah O’Connell, Aakash Patel, Erwin Rosales-Zavala, Elizabeth Scheideman, Nicole A. Schneck, Zachary Schneiderman, Andrew Shaddeau, William Shadrick, Alison Vinitsky, Sara Witter, Yanhong Yang, and Yaqiu Zhang.

## STAR★Methods

### Key resources table

**Table:** 

REAGENT or RESOURCE	SOURCE	IDENTIFIER
**Antibodies**		

RM19R	Cottrell et al.^4^	N/A
RM20A2	Cottrell et al.^4^	N/A
RM19B1	Cottrell et al.^4^	N/A
RM20G	Cottrell et al.^4^	N/A
VRC01	Wu et al.^32^http://www.hiv.lanl.gov/	RRID: AB_2491019
PGT145	Lee et al.^33^	RRID: AB_2491054
CAP256-VRC26.25	Doria-Rose et al.^34^	N/A
PGT151	Blattner et al.^35^	N/A
VRC34.01	Kong et al.^36^	RRID: AB_2819225
VRC-PG05	Zhou et al.^37^	N/A
Motavizumab	Thermo Fisher Scientific	Cat#: MA5-41713; RRID: AB_2910856
2G12	Trkola et al.^38^	RRID: AB_2819235
447-52D	Sharon et al.^39^	RRID: AB_2491016
3074	Hioe et al.^40^	N/A
CR9114	Dreyfus et al.^41^	N/A
F105	Posner et al.^42^	N/A
35O22	Huang et al.^43^	N/A
8ANC195	Scharf et al.^44^	N/A

**Bacterial and virus strains**		

BG505	Wu et al.^45^	N/A
BG505Δ611	Kong et al.^36^	N/A
10-strain panel for neutralization assessments	Xu et al.^11^	N/A

**Chemicals, peptides, and recombinant proteins**		

Superdex200 10/300GL Column	GE Healthcare Life Sciences	Cat#: 28990944
MabSelect SuRe Protein A Resin	GE Healthcare Life Sciences	Cat#: 17543802
KLH	ThermoFisher Scientific Inc.	Cat#: 77600
MBS (m-maleimidobenzoyl-N-hydoxysuccinimide ester)	ThermoFisher Scientific Inc.	Cat#: 22311
Sodium thiocyanate (NaSCN)	Sigma	Cat#: 80518-100ML-F, lot: #BCBR4380V
Aluminum Hydroxide	Leidos Biomed	Cat#: D-15-0017
Adjuplex	Sigma-Aldrich Inc, MO or Adjuplex equivalent formulated based on US Patent 6,676,958 B2	Cat#: HRW-1560-49
Iscomatrix	IAVI	CSL Lot XR004
Matrix M	Novavax	Cat#: M1-104
GLA-LSQ	IDRI	Cat#: QG806
TQL-1055	AMRI	Cat#: WZH-AF-168-1
Cysteine-added FP8 peptide: AVGIGAVFC	Xu et al.^11^	N/A
Cysteine-added FP7 peptide: AVGIGAVC	Xu et al.^11^	N/A
Cysteine-added FP6 peptide: AVGIGAC	Xu et al.^11^	N/A
BG505 DS-SOSIP	Kwon et al.^15^	N/A
BG505 DS-SOSIPAvi	Kong et al.^36^	N/A
CH505 DS-SOSIP FP degly3	Cheng et al.^8^	N/A
CH505 DS-SOSIP FP degly4	Cheng et al.^8^	N/A
CH505 DS-SOSIP CD4bs degly	Cheng et al.^8^	N/A
FP8v1-KLH	Xu et al.^11^	N/A
FP8v1-rTTHC-EN-nanoparticle	This paper	N/A
glycan-base BG505 DS-SOSIP trimer	Olia et al.^21^	N/A
BG505 DS-SOSIP JG2 trimer	This paper	N/A

**Critical commercial assays**		

Turbo293™ Transfection Kit	ThermoFisher Scientific Inc.	Cat#: A14525
BirA biotin-protein ligase bulk reaction kit	Avidity	Cat#: BirA500
Human CXCL13/BLC/BCA-1 Quantikine ELISA Kit	bio-techne/R&D systems	Cat#: DCX130
MSD 96-well bare plates	MSD	Cat#: L15XA-3
SULFO-TAG	MSD	Cat#: R91AO-1
SureBlue tetramethylbenzidine (TMB) substrate	KPL	Cat#: 5120-0075

**Experimental models: Cell lines**		

Expi293F cells	ThermoFisher Scientific Inc	Cat#: A14527
FreeStyle 293-F cells	ThermoFisher Scientific Inc	Cat#: R79007

**Experimental models: Organisms/strains**		

Indian origin rhesus macaque	Corrigan et al.^20^	N/A

**Recombinant DNA**		

pVRC8400 vector	https://www.addgene.org	Cat# 63160
pVRC8400-RM19R plasmid	Corrigan et al.^20^	N/A
pVRC8400-RM20A2 plasmid	Corrigan et al.^20^	N/A
pVRC8400-RM19B1 plasmid	Corrigan et al.^20^	N/A
pVRC8400-RM20G plasmid	Corrigan et al.^20^	N/A

**Software and algorithms**		

GraphPad Prism Software	GraphPad Prism Software, Inc.	N/A
The PyMol Molecular Graphics System, v2	Schrödinger, LLC	https://pymol.org/2/
BLI Acquisition & Analysis Software	ForteBio	https://www.fortebio.com/products/octet-systems-software
EPU	ThermoFisher Scientific	https://www.thermofisher.com/us/en/home/electron-microscopy/products/software-em-3d-vis/epu-software.html
EMAN2.1	Tang et al.^46^	https://blake.bcm.edu/emanwiki/EMAN2
RELION	Scheres^47^	https://www3.mrc-lmb.cam.ac.uk/relion/index.php/Main_Page
SPIDER	Shaikh et al.^48^	https://spider.wadsworth.org/spider_doc/spider/docs/spider.html
UCSF Chimera	Pettersen et al.^49^	https://www.cgl.ucsf.edu/chimera/
FREALIGN	Grigorieff^50^; Lyumkis et al.^51^	https://grigoriefflab.umassmed.edu/frealign

### Resource availability

#### Lead contact

Further information should be directed to and will be fulfilled by Peter D. Kwong (pdkwong@nih.gov).

#### Materials availability

Requests for resources and reagents should be directed to and will be fulfilled by Theodore C. Pierson (piersontc@niaid.nih.gov) or Peter D. Kwong (pdkwong@nih.gov). All new reagents are available by MTA for non-commercial research.

#### Data and code availability

• The published article includes all data generated or analyzed during this study.
• This study did not generate new code.
• Any additional information required to reanalyze the data reported in this paper is available from the lead contact upon request.

### Experimental model and study participant details

#### NHP studies

Local, state, federal and institute policies were implemented and maintained while caring for the animals involved in this study in an American Association for Accreditation of Laboratory Animal Care-accredited facility (Bioqual Inc, MD). The Animal Care and Use Committee of the Vaccine Research Center, NIAID, NIH approved each of the studies involved, which included those listed under protocols VRC #16-666.1 for the CH505 DS-SOSIP CD4bs deglycosylated groups, #16-667.2 for the FP primed groups, #16-450.5 for the short-interval adjuvant study, and #16-667.4 for the long-interval adjuvant study. Healthy (B-virus, SIV, SRV, and STLV negative) Indian rhesus macaques without previous exposure to HIV or SHIV and without prior involvement in procedures or drug experimentation of both sexes, aged 2-14 years, and with body weights ranging from 41-109kg were evenly distributed to different groups in each of the studies based on body weights. For immunization, 100-200 μg of specified, filter-sterilized immunogen mixed in a total volume of 1ml with PBS and specified adjuvant was injected at 500 μl to the caudle thigh of the two hind legs for I.M. or S.C. immunization, and. Two weeks after each immunization, whole blood was collected, and plasma and peripheral blood mononuclear cells (PBMCs) were isolated using Ficoll density gradient centrifugation.

#### Adjuvants

For each NHP immunization, 200 μl of Adjuplex (Sigma-Aldrich Inc, MO or Adjuplex equivalent formulated based on US Patent 6,676,958 B2), 50 μg of Matrix M (Novavax), 500 μg of Aluminum Hydroxide (Leidos Biomed, Frederick, MD), 75 U Iscomatrix (IAVI), 25 μg of GLA-LSQ, 200 μg of TQL-1055 (Adjuvance Technologies, Inc) was mixed with immunogen for adjuvant studies.

#### Cell lines

Expi293F cells (cat# A14257) and FreeStyle 293-F cells (cat# R79007) were purchased from ThermoFisher Scientific Inc. Cells were maintained in FreeStyle 293 Expression Medium. The cell line was used directly from the commercial sources and cultured following manufacturer suggestions as described in Method details below.

### Method details

#### Base-binding antibody and Fab production

The RM19R heavy variable region was synthesized with a human IgG1 heavy constant backbone for expression of mAb. Heavy and light chain pairs were co-transfected (1:1 mass ratio) in Expi293F cells (Thermo Fisher) using ExpiFectamine 293 according to the manufacturer’s protocol (Thermo Fisher). Six days post-transfection, culture supernatants were harvested.^4^^,^^20^

#### Base-binding BG505 DS-SOSIP competition ELISA for NHP samples

The competition ELISA using the base-specific Fab RM19R was carried out as previously described. Briefly, following overnight incubation of Costar half plates with 2 μg/ml Lectin and a one-hour blocking step with 5% skim milk/PBS, BG505 DS-SOSIP was incubated on the plates for two hours at RT. After washing the plates 5x with PBS-T, RM19R at 2 μg/ml or PBS was incubated on half of the plates for one hour at RT. Without washing, serially diluted NHP sera at various starting dilutions was added to the plates and further incubated for one hour at RT. The plates were then washed 5x with PBS-T and goat anti-NHP HRP-conjugated secondary antibody was added to the plates at a 1:5000 dilution for one hour at RT. The plates were then washed and TMB substrate was used to develop the plates for 10 minutes, followed by the addition of H_2_SO_4_ to stop the reaction. The plates were then read at OD450nm, and values were documented.

#### Glycan-base trimer ELISA for NHP samples and monoclonal antibodies

The ELISA using the glycan-base BG505 DS-SOSIP trimer was carried out similarly as previously described with BG505 DS-SOSIP trimer.^20^ Briefly, following overnight incubation of Costar half plates with 2 μg/ml Lectin and a one-hour blocking step with 5% skim milk/PBS, the glycan-base trimer was incubated on the plates for two hours at RT. After washing the plates 5x with PBS-T, serially diluted NHP sera at various starting dilutions or monoclonal antibodies at 2 μg/ml was added to the plates and further incubated for one hour at RT. The plates were then washed 5x with PBS-T and goat anti-NHP HRP-conjugated secondary antibody was added to the plates at a 1:5000 dilution for one hour at RT. The plates were then washed and TMB substrate was used to develop the plates for 10 minutes, followed by the addition of H_2_SO_4_ to stop the reaction. The plates were then read at OD450 nm and values were documented.

#### BG505 DS-SOSIP avidity ELISA assay with sodium thiocynate (NaSCN)

The avidity test of NHP plasma samples to BG505 DS-SOSIP is similar to the glycan-base trimer ELISA, with duplicates of NHP plasma added to left and right halves of 96-well plates coated with BG505 DS-SOSIP trimer. After incubation for 1h at RT, the plates were washed with 5x with PBS-T, then 3M NaSCN (Sigma, 80518-100ML-F, lot: #BCBR4380V) was added for 15 minutes at RT to one half of the plate, and PBS was added to the other half of the plate, then plates were washed 5x with PBS-T, and goat anti-NHP HRP-conjugated secondary antibody was added to the plates. The reduction with NaSCN was calculated by taking the difference between AUC values without and with NaSCN divided by the AUC values without NaSCN and multiplied by 100.

#### Anti-Fusion-Peptide (FP8) ELISA

The ELISA for assessing the response to Fusion-Peptide (FP8) was conducted with 96-well streptavidin-coated plates (Thermo Fisher) and biotinylated eight-residue fusion peptide (FP8-PEG-biotin). Subsequently, the plates were blocked with B3T buffer (comprising of 150 mM NaCl, 50 mM Tris-HCl, 1 mM EDTA, 3.3% fetal bovine serum, 2% bovine albumin, 0.07% Tween 20, and 0.02% thimerosal) prepared in-house. The serum was serially diluted at 7-point 5-fold dilution and incubated for 1hr. Goat anti-NHP HRP conjugated secondary was added and incubated for an hour. The plates were then developed for 10 minutes using tetramethylbenzidine (TMB) substrate (SureBlue; KPL, Gaithersburg, MD). To stop the reaction, 1 N H_2_SO_4_ sulfuric acid was added. Finally, the plates were read on a microplate spectrophotometer (Biotek Epoch, Winooski, VT) to determine the endpoint titer for each sample.^8^

#### Neutralization assays

Neutralization assays using a single round of infection Env-pseudovirus were performed by using TZM-Bl target cells and heat inactivated NHP sera.^8^^,^^10^ Briefly, 293T cells co-transfected with an Env expression plasmid and a sPG3ΔEnv backbone were used to generate the Env-pseudovirus stocks used in the assay. The sera were assessed at various dilutions, using an 8-point 4-fold dilution method which began at a dilution factor of 1:20. The 50% inhibitory dilutions (ID50) were used to assess breadth across the 10 FP-sensitive HIV-1 strains and to test for any correlative relationship with the plasma and B cell parameters tested.

#### FP-KLH immunogens

FP8 (AVGIGAVF), FP7 (AVGIGAV) and FP6 (AVGIGA) were synthesized (GenScript) with the addition of a Cysteine at the C terminus and conjugated to m-maleimidobenzoyl-N-hydroxysuccinimide ester (MBS) activated keyhole limpet hemocyanin (KLH) (Thermo-Scientific).^10^^,^^11^ The FP-KLH conjugates were assessed antigenically for binding to FP-specific antibodies VRC34.01, PGT151 and ACS202 with Biolayer Interferometry on an Octet RED384 (ForteBio) instrument.^8^

#### HIV-1 envelope trimer immunogens

All HIV-1 envelope trimer immunogens were prepared using transiently transfected 293F cells.^1^^,^^52^ Broadly neutralizing antibodies 2G12 or VRC01 were used in affinity chromatography to purify the trimers, along with gel filtration (Superdex200 16/60HL column) and a 447-52D affinity column functioning as a negative selection to remove V3-exposed trimers. Antigenicity tests were performed on the trimers with or without mixing with adjuvant using a Meso Scale Discovery (MSD),^15^ as described below.

#### Meso scale discovery assay

Antigenicity tests were performed on BG505 DS-SOSIP trimer mixed with different adjuvants using a Meso Scale Discovery (MSD) platform.^27^ MSD 96-well bare plates (Cat# L15XA-3) were coated with anti-HIV broadly neutralizing monoclonal antibodies at a coating concentration of 4 μg/mL for 16-24 hours at 4°C. Following the overnight incubation, plates were washed and blocked in MSD Blocker A for 1 hour, shaking at room temperature. Following blocking, plates were washed and incubated with serially diluted HIV trimer (starting concentration of 5 μg/mL and seven two-fold serial dilutions) for 2 hours shaking at room temperature. Plates were then washed and 5 μg/mL SULFO-TAG (MSD, cat #R91AO-1)-labelled 2G12 secondary antibody was added, shaking at room temperature for 1 hour. After the detection antibody incubation, plates were washed, 1X MSD Read Buffer was applied, and plates were analyzed using the MSD Sector Imager S600. All samples were tested in duplicate. Serial dilutions of sample were used to calculate and assign an area under the curve (AUC) value as the primary readout. Results were plotted and analyzed using Prism version 8 or newer (GraphPad, San Diego, CA). Samples with a replicate coefficient of variation > 30% were retested.

#### CXCL13 quantification in NHP plasma

CXCL13 levels in NHP plasma were measured using Human CXCL13/BLC/BCA-1 Quantikine ELISA Kit (bio-techne/R&D systems) following instruction, with Human CXCL13 protein at 2-fold dilutions start at 500pg/ml as the standard for quantification.

### Quantification and statistical analysis

For two group comparison analysis of serological and B cell responses, a two-tailed Mann-Whitney t-tests comparing the mean and standard error of the mean (SEM) was performed. For ELISA, the percentage of base BG505 DS-SOSIP response was calculated by taking the difference between the AUC values or OD values at indicated dilutions of the wells receiving blocking buffer only and RM19R Fab, divided by the ACU or OD values of the wells with receiving blocking buffer only. For multiple comparison analysis for adjuvant studies, Kruskal-Wallis test followed with two-stage linear step-up procedure of Benjamini, Krieger and Yekutieli tests were performed to assess p values between different adjuvant groups. Two-tailed Pearson correlation coefficient test was used to assess correlations between plasma and B cell parameters and neutralization titers and breadth, if data obeys normality assumptions. If normality assumption fails, then correlation test was performed with 2-tailed Spearman correlation coefficient test. ∗: p<0.05, ∗∗: p<0.01, ∗∗∗: p<0.0001.
