# Supplementary material for: Long trimer-immunization interval and appropriate adjuvant reduce immune responses to the soluble HIV-1-envelope trimer base
Source: iScience. 2024 Jan 11;27(2):108877. doi: 10.1016/j.isci.2024.108877 (PMC10839646; doi:10.1016/j.isci.2024.108877)
Supplement: Document S1. Figures S1–S7 [file mmc1.pdf]

iScience, Volume *27*

## Supplemental information

# Long trimer-immunization interval and appropriate adjuvant reduce immune responses to the soluble HIV-1-envelope trimer base

**Hongying Duan, Angela R. Corrigan, Cheng Cheng, Andrea Biju, Christopher A. Gonelli, Adam S. Olia, I-Ting Teng, Kai Xu, Sijy O'Dell, Sandeep Narpala, Mike Castro, Leonid Serebryannyy, Jennifer Wang, Danealle K. Parchment, Edward K. Sarfo, Jelle van Schooten, John-Paul Todd, Shuishu Wang, Darcy R. Harris, Hui Geng, Alexander J. Jafari, VRC Production Program, Ruth A. Woodward, Nicole A. Doria-Rose, Kathryn E. Foulds, Adrian B. McDermott, Marit J. van Gils, Richard A. Koup, Theodore C. Pierson, Peter D. Kwong, and John R. Mascola**

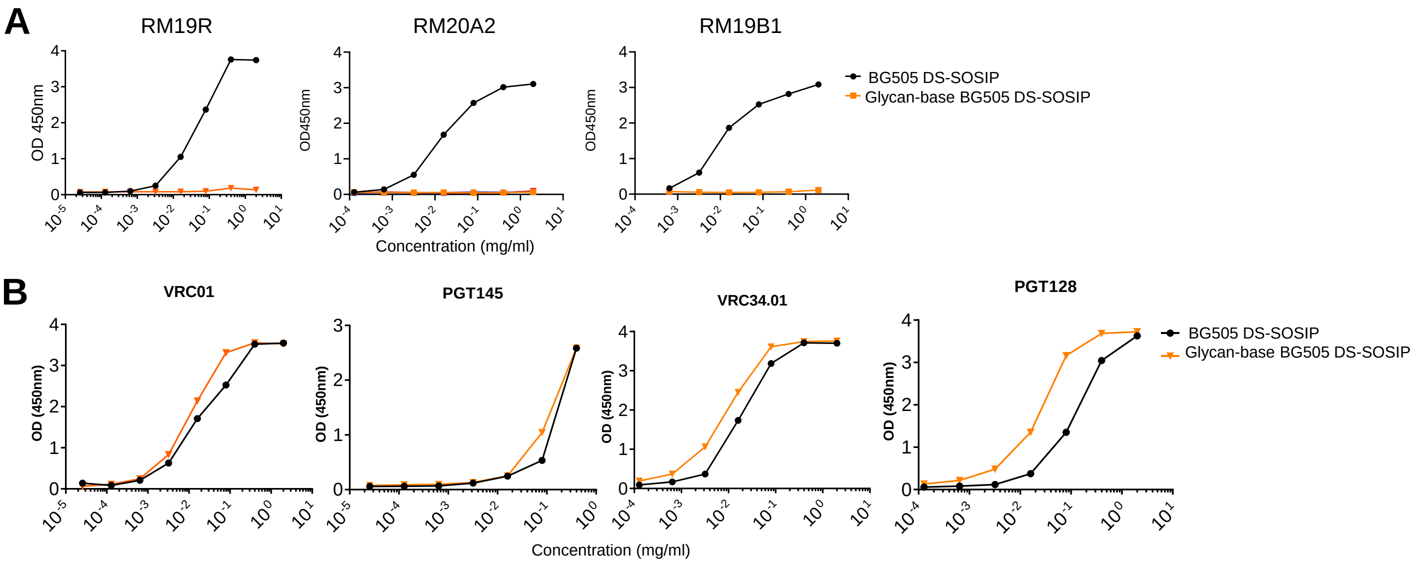


**Figure S1. Glycan-base BG505 DS-SOSIP trimer does not show binding to base-specific mAbs, but shows similar or higher binding to bNAbs, related to Figures 1, 3, 6, 7.** (**A**) Total trimer response as measured by ELISA OD450nm using either BG505 DS-SOSIP (black) or glycan-base BG505 DS-SOSIP trimer (orange) with base-specific mAbs RM19R (left panel), RM20A2 (middle panel), and RM19B1 (right panel), and (**B**) with epitope-specific bNAbs VRC01 (left panel), PGT145 (second panel from left), VRC34.01 (second panel from right), and PGT128 (right panel).

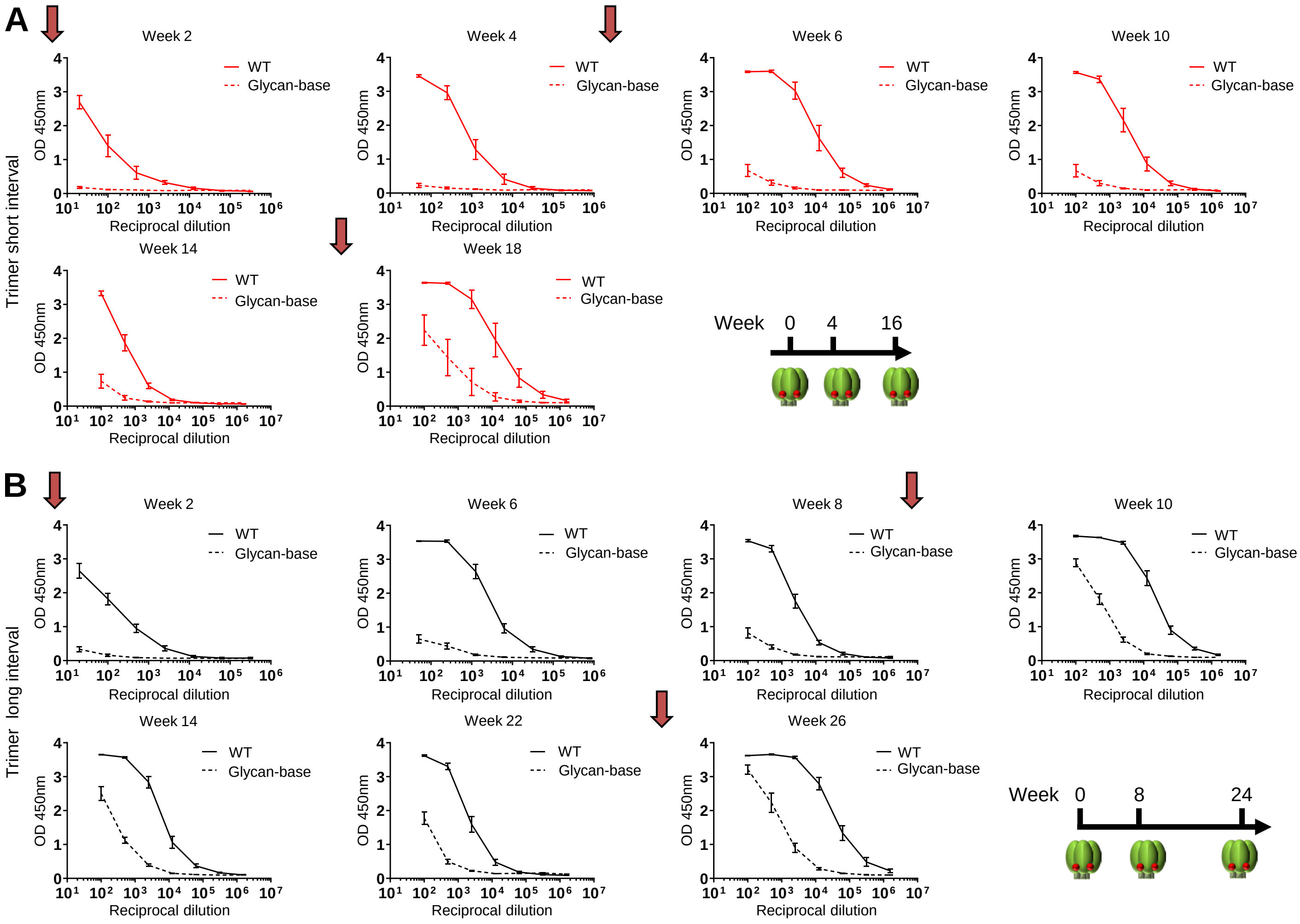


**Figure S2. ELISA with additional timepoints demonstrate that the long interval between the first and second trimer immunization is critical for enhanced non-base trimer responses, related to Figure 3.** (**A**) Plasma binding to BG505 DS-SOSIP (solid line) and glycan-base BG505 DS-SOSIP (dashed line) in NHPs with short interval (I.M.) at week 2, 4, 6, 10, 14, and 18. (**B**) Plasma binding to BG505 DS-SOSIP (solid line) and glycan-base BG505 DS-SOSIP (dashed line) in NHPs with long interval (S.C.) at weeks 2, 6, 8, 10, 14, 22, and 26. Red arrows indicate BG505 trimer immunizations at week 0,4,16 for short interval NHPs in (**A**) and at week 0, 8, 24 for long interval NHPs in (**B**).

**A**

| | | Sample | BG505.W6M.C2.T332N.SG3 | MW965.26.SG3 | SIVmac251.30.SG3 | Anti-V3 peptide (ET) | Anti-FP8 peptide (ET) |
|---|---|---|---|---|---|---|---|
| | ID50 | Clade | A | C | non HIV | | |
| | | | week 18/26 | week 18/26 | week 18/26 | week 18/26 | week 18/26 |
| 1 | week 0,4,16 week 18 | A12V183 | 15 | 50 | <10 | 7500 | |
| | | 13N001 | 129 | 51 | <10 | 2500 | |
| | | A12V185 | <10 | <10 | <10 | 500 | |
| | | A12V030 | <10 | 35 | <10 | 500 | |
| | | A12V093 | 30 | 48 | <10 | 1500 | |
| 2 | week 0,8,24 week 26 | DGTT | <20 | 318 | <20 | 1500 | 300 |
| | | DGKV | <20 | 84 | <20 | 1500 | 2500 |
| | | DGGP | <20 | <20 | <20 | 2500 | 1500 |
| | | HZ8 | 75 | 336 | <20 | 7500 | 500 |
| | | A12V150 | <20 | 42 | <20 | 7500 | 2500 |
| | | 14PP01 | <20 | 237 | <20 | 1500 | 100 |

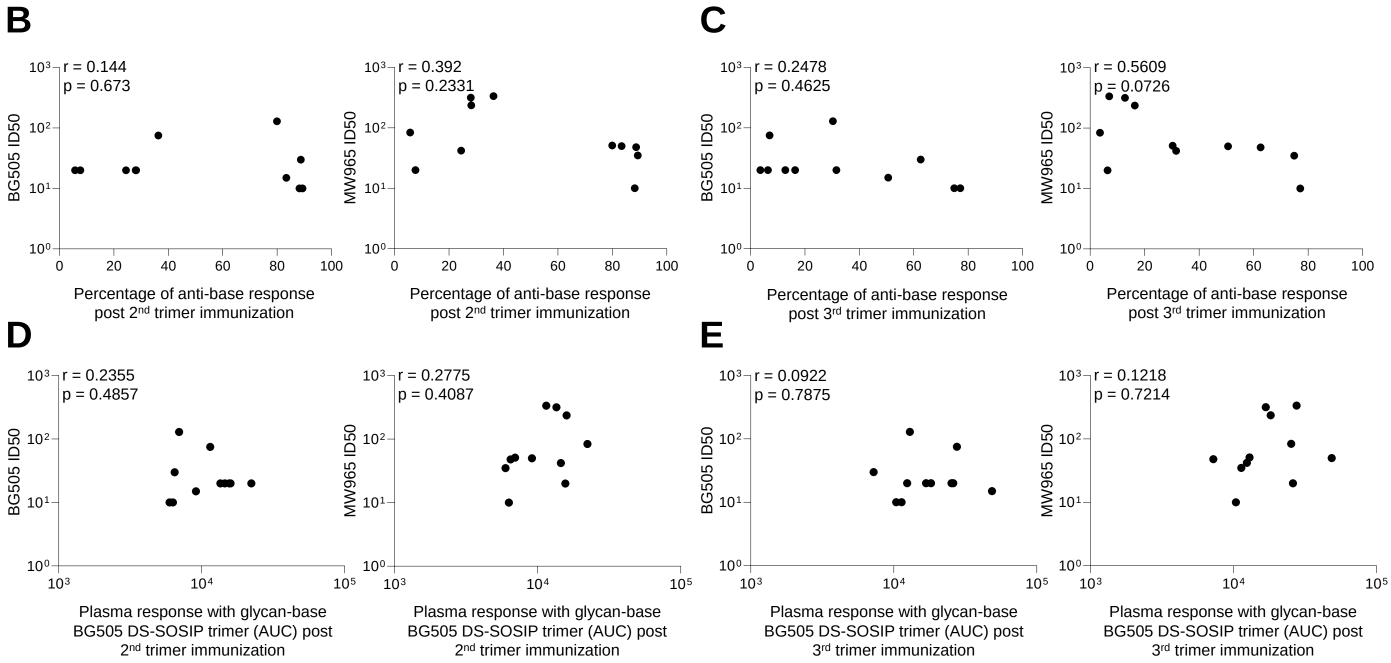


**Figure S3. In trimer only immunized NHPs, anti-base responses do not correlate with neutralizing activity, related to Figure 3.** (**A**) Plasma neutralizing ID50 titers and anti-BG505 v3 peptide endpoint titers at week 18 for short-interval and week 26 for long-interval NHPs, both two weeks post the third trimer immunization. (**B, C**) Correlation between anti-base response post second (**B**) or third (**C**) trimer immunization with neutralizing ID50 titers on BG505 (left) or MW965 (right). (**D, E**) Correlation between plasma response to glycan-base BG505 DS SOSIP trimer with neutralizing ID50 titers on BG505 (left) or MW965 (right) post second (**D**) or third (**E**) trimer immunization. Correlation analysis was performed using a two-tailed Pearson correlation coefficient test.

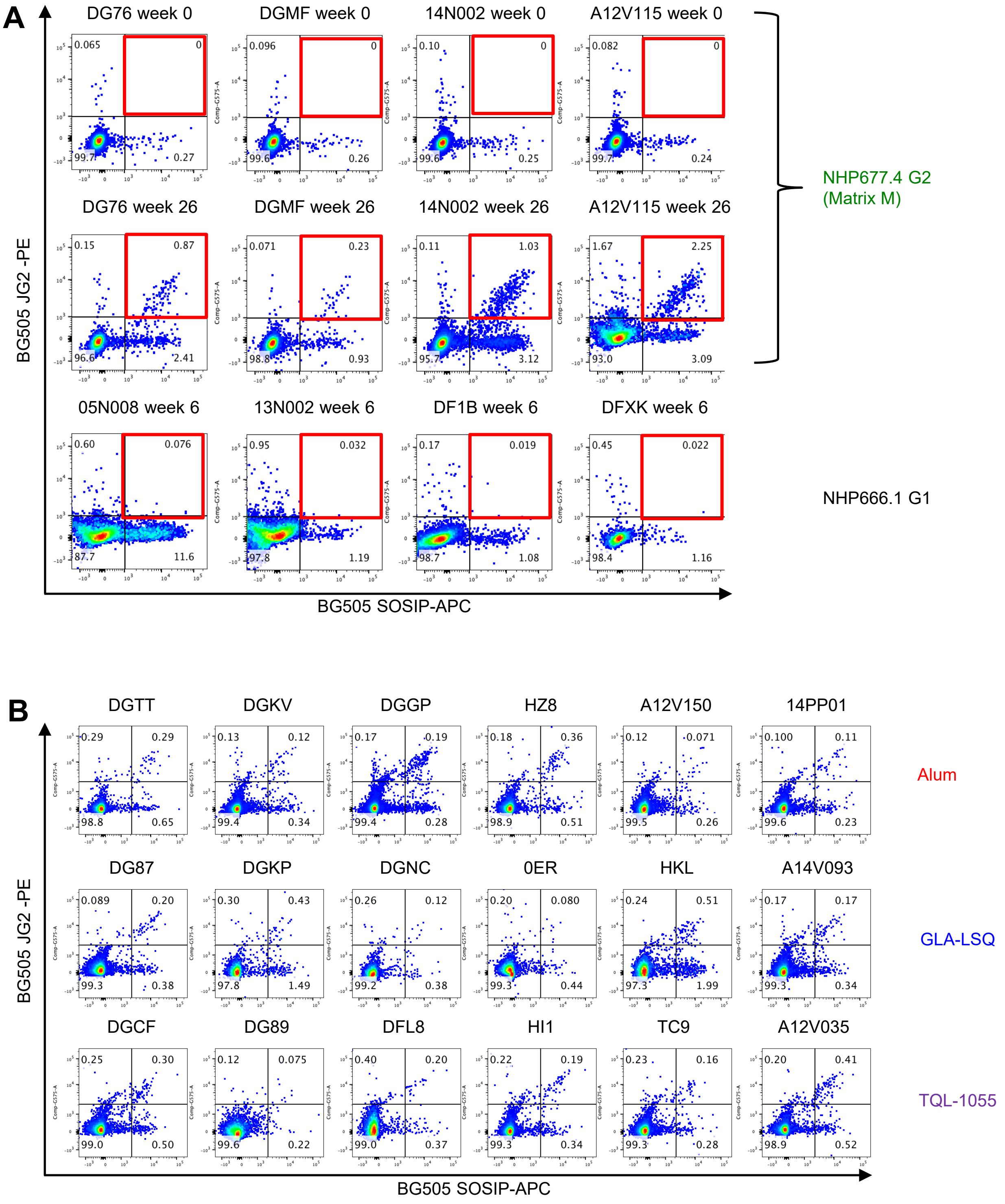


**Figure S4. PBMC analysis using BG505 DS-SOSIP trimer and glycan-base BG505 DS-SOSIP trimer (BG505 JG2) probes, related to Figures 5 and 6.** (**A**) PBMCs of NHP677.4 G2 at pre immunization week 0 and post 3rd trimer immunization week 26, compared to PBMCs from NHP666.1 G1 at week 6. (**B**) PBMCs from the other three NHP 677.4 groups at post 3rd trimer immunization week 26.

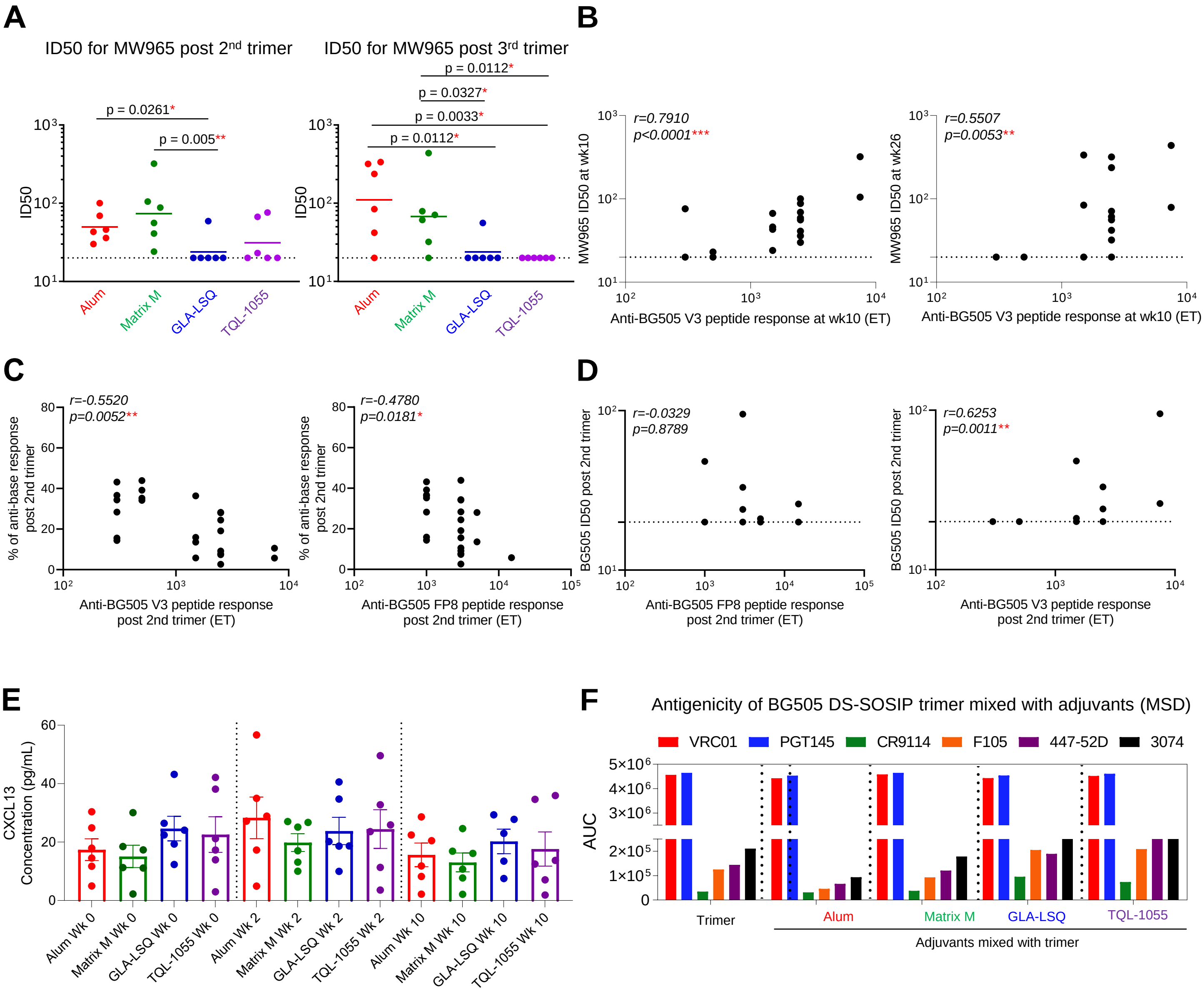


**Figure S5. Neutralizing and binding titers at week 10 and week 26 for NHP677.4 study, related to Figure 6.** (**A**) Geometric mean ± 95% CI are shown for neutralizing ID50 titers for week 10 and week 26 plasma on MW965. (**B**) Correlation between plasma anti-V3 peptide response (endpoint titers at week 10) and neutralizing titers for MW965 (**F**) at week 10 (left panel) and week 26 (right panel). (**C**) Correlation between plasma anti-V3 peptide response (endpoint titers at week 10, left panel) and plasma anti-FP peptide response (endpoint titers at week 10, right panel) with percentages of anti-base response at week 10. (**D**) Correlation between plasma anti-FP peptide response (left panel), anti-V3 peptide response (right panel) and BG505 ID50 at week 10. (**E**) CXCL13 concentrations in immunized NHP plasma at week 0, week 2, and week 10. (**F**) Antigenicity of BG505 DS-SOSIP trimer mixed with adjuvants (MSD). Data are shown with mean ± SEM for multiple comparison analysis, with Kruskal-Wallis test followed with two-stage linear step-up procedure of Benjamini, Krieger and Yekutieli tests to assess p values between different adjuvant groups. *: p<0.05; **: p <0.01; ***: p <0.001; ****: p <0.0001 ; ns, not significant. (**A**). Correlation analysis was performed using a two-tailed Pearson correlation coefficient test for (**B**-**D**). *: p<0.05; **: p <0.01; ***: p <0.001; ****: p <0.0001.

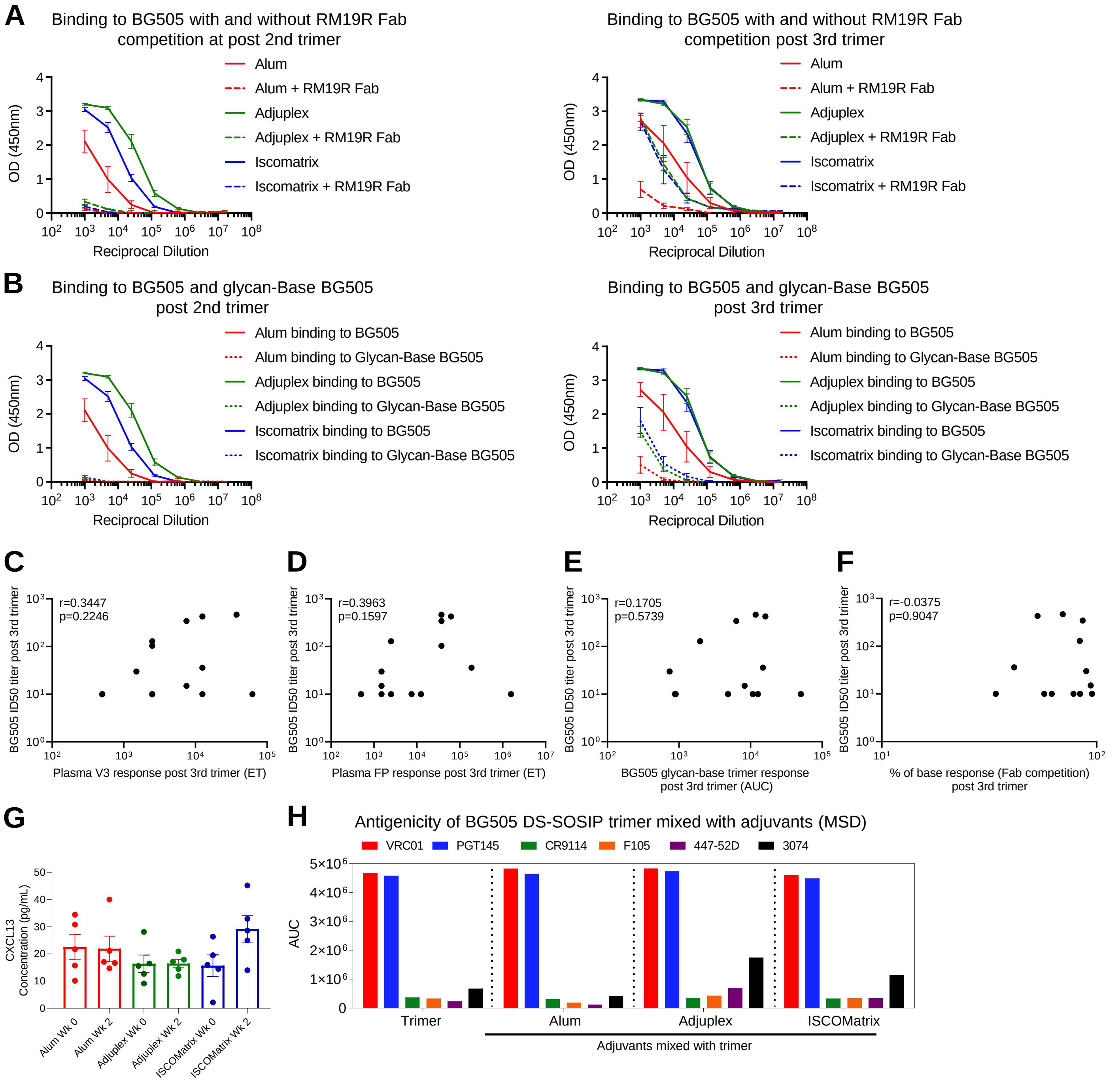


**Figure S6. In short-interval trimer immunization, adjuvants impact plasma anti-base response, but do not impact autologous neutralizing titers, related to Figure 7.** (**A**) OD curves of total BG505 DS-SOSIP trimer response and non-base region directed response measured via competition with RM19R Fab at week 6 and week 18, two weeks post the second and third trimer immunization. (**B**) OD curves of total BG505 DS-SOSIP trimer response and non-base region directed response measured via glycan-base BG505 DS-SOSIP trimer (**C**) Correlation between V3 and BG505 ID50 titers at week 18. (**D**) Correlation between FP response measured via ELISA endpoint titer and BG505 neutralization titers. (**E**) Correlation between glycan-base BG505 response measured via ELISA endpoint titer and BG505 neutralization titers. (**F**) Correlation between % of base response measured via RM19R Fab competition assay and BG505 neutralization titers. (**G**) CXCL13 concentration in immunized NHP plasma at week 0 and week 2. (**H**) Antigenicity of BG505 DS-SOSIP trimer mixed with adjuvants (MSD). Two-tailed Mann-Whitney nonparametric tests were used for statistical analysis to assess p values for mean ± SEM. *: $p<0.05$; **: $p <0.01$; ***: $p <0.001$; ****: $p <0.0001$ ; ns, not significant. The Data in A-B represent 2 independent experiments with similar results. Correlation analysis was performed using a two-tailed Spearman correlation coefficient test.

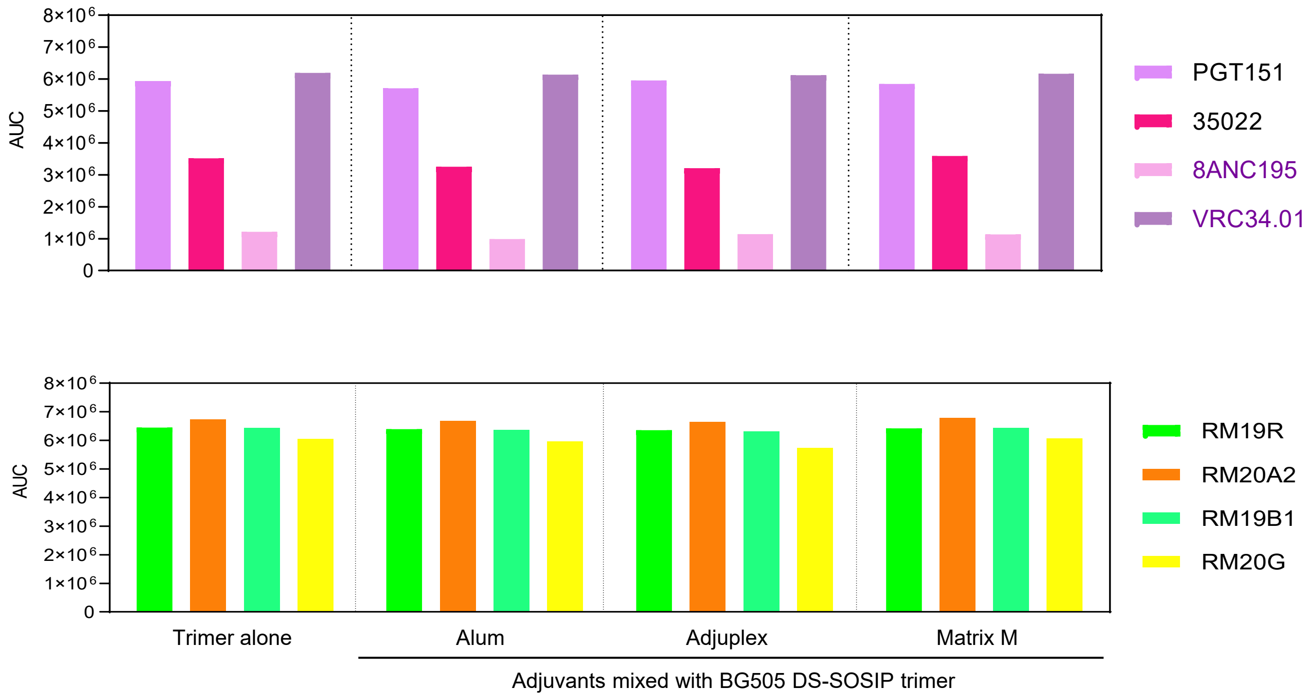


**Figure S7: Adjuvants do not change antigenicity of BG505 DS-SOSIP trimer at FP, gp120/gp41 interface or base region, related to Figure 6 and 7.** BG505 DS-SOSIP trimer mixed with PBS (trimer alone), Alum, Adjuplex or Matrix M were detected with anti-FP, anti-gp120/41 interface mAbs (top), and anti-base mAbs (bottom), as determined by AUC from MSD assay.
